# Elderberry for prevention and treatment of viral respiratory illnesses: a systematic review

**DOI:** 10.1186/s12906-021-03283-5

**Published:** 2021-04-07

**Authors:** L. Susan Wieland, Vanessa Piechotta, Termeh Feinberg, Emilie Ludeman, Brian Hutton, Salmaan Kanji, Dugald Seely, Chantelle Garritty

**Affiliations:** 1 Center for Integrative Medicine, University of Maryland School of Medicine, Baltimore, MD USA; 2 Evidence-based Oncology, Department I of Internal Medicine, Center for Integrated Oncology Aachen Bonn Cologne Duesseldorf, Faculty of Medicine and University Hospital Cologne, University of Cologne, Cologne, Germany; 3 Kelly Government Solutions, Rockville, MD USA; 4 Pain Research, Informatics, Multimorbidities, and Education (PRIME) Center, VA Connecticut Healthcare System, West Haven, CT USA; 5 Yale Center for Medical Informatics, Yale University School of Medicine, New Haven, CT USA; 6 University of Maryland, Health Sciences and Human Services Library, Baltimore, MD USA; 7 The Ottawa Hospital Research Institute (OHRI), Ottawa, Ontario Canada; 8 University of Ottawa, School of Epidemiology and Public Health, Ottawa, Ontario Canada; 9 The Ottawa Hospital, Ottawa, Ontario Canada; 10 The Canadian College of Naturopathic Medicine, Toronto, Ontario Canada; 11 The Centre for Health Innovation, The Canadian College of Naturopathic Medicine, Ottawa, Ontario Canada

**Keywords:** Sambucus, Elderberry, Viral illness, Respiratory illness, Inflammation, Cytokines, COVID-19, Systematic review

## Abstract

**Background:**

Elderberry has traditionally been used to prevent and treat respiratory problems. During the COVID-19 pandemic, there has been interest in elderberry supplements to treat or prevent illness, but also concern that elderberry might overstimulate the immune system and increase the risk of ‘cytokine storm’. We aimed to determine benefits and harms of elderberry for the prevention and treatment of viral respiratory infections, and to assess the relationship between elderberry supplements and negative health impacts associated with overproduction of pro-inflammatory cytokines.

**Methods:**

We conducted a systematic review and searched six databases, four research registers, and two preprint sites for studies. Two reviewers independently assessed studies for inclusion, extracted data from studies, assessed risk of bias using Cochrane tools, and evaluated certainty of estimates using GRADE. Outcomes included new illnesses and the severity and duration of illness.

**Results:**

We screened 1187 records and included five randomized trials on elderberry for the treatment or prevention of viral respiratory illness. We did not find any studies linking elderberry to clinical inflammatory outcomes. However, we found three studies measuring production of cytokines ex vivo after ingestion of elderberry. Elderberry may not reduce the risk of developing the common cold; it may reduce the duration and severity of colds, but the evidence is uncertain. Elderberry may reduce the duration of influenza but the evidence is uncertain. Compared to oseltamivir, an elderberry-containing product may be associated with a lower risk of influenza complications and adverse events. We did not find evidence on elderberry and clinical outcomes related to inflammation. However, we found evidence that elderberry has some effect on inflammatory markers, although this effect may decline with ongoing supplementation. One small study compared elderberry to diclofenac (a nonsteroidal anti-inflammatory drug) and provided some evidence that elderberry is as effective or less effective than diclofenac in cytokine reduction over time.

**Conclusions:**

Elderberry may be a safe option for treating viral respiratory illness, and there is no evidence that it overstimulates the immune system. However, the evidence on both benefits and harms is uncertain and information from recent and ongoing studies is necessary to make firm conclusions.

**Supplementary Information:**

The online version contains supplementary material available at 10.1186/s12906-021-03283-5.

## Background

In 2019, the novel severe acute respiratory syndrome coronavirus 2 (SARS-CoV-2) emerged in human populations. The virus proved to be transmissible between humans and led to a global pandemic of coronavirus disease 2019 (COVID-19). The public has sought various alternative and complementary therapies to support prevention and treatment of COVID-19 [1].

One popular complementary health approach to preventing and treating illness is the use of over-the-counter herbal supplements. Various parts of the elderberry plant (Sambucus spp.) have historically been used both as foods and as remedies for health problems [2, 3]. Specifically, the flowers and dried or cooked fruit (‘elderflower’ and ‘elderberry’, respectively) have traditionally been used for respiratory problems such as colds and influenza [4–6]. Many people in the United States consume herbal dietary supplements with a belief that they offer safe and effective options to help them maintain health and wellness, and elderberry herbal supplement sales nearly doubled in the United States between 2017 and 2018 [7]. A recent systematic review looked at elderberry for the treatment of upper respiratory symptoms and suggested that elderberry could be helpful in shortening the duration of colds or influenza [8]. While herbal supplements including elderberry have been marketed to boost immunity from respiratory illness, the U.S. Federal Drug Administration (FDA) has issued warning letters to supplement producers who are marketing such products with claims that they prevent, treat, or cure COVID-19, as this has not been proven [9].

Elderberry contains anthocyanins, a subset of flavonoids which may have immunomodulating and possibly anti-inflammatory effects [10]. Anthocyanins can attach to (and render ineffective) viral glycoproteins that enable viruses to enter host cells, thereby potentially having an inhibitory effect on viral infection. Extracts of elderberry have demonstrated in-vitro to have inhibitory effects on influenza A and influenza B viruses [11, 12] as well as H1N1 “swine” flu virus [13]. In addition to this direct action on viruses, elderberry may have an effect on the immune system through cytokines. There is some evidence that elderberry increases the production of inflammatory cytokines (i.e., TNF-alpha, interleukins) although there is also evidence suggesting that cytokine production is decreased [12, 14, 15]. In some cases of COVID-19, proinflammatory cytokines are overproduced and the patient experiences what has been called a ‘cytokine storm’ (when the cytokines begin to attack the cells and tissues of the body) which increases risk of multiorgan failure and death [16, 17]. There is therefore concern in COVID-19 that the potential benefits of elderberry in inhibiting viral replication may be negated by potential harms from cytokine release and immune system hyperresposiveness [18]. The 2019 systematic review predates COVID-19 and did not look at elderberry for prevention of illness, did not allow inclusion of mixed elderberry products, and did not include the effects of elderberry upon clinical or ex vivo cytokine-related outcomes. We therefore decided to use Cochrane systematic review methods to re-assess the current evidence on the potential benefits and harms of elderberry for prevention or treatment of viral respiratory illness.

## Methods

The protocol was registered with PROSPERO on 11 June 2020 and is available from https://www.crd.york.ac.uk/prospero/display_record.php?ID=CRD42020189959.

### Study objectives

We conducted a systematic review of studies with the following aims:

1. To assess the benefits and harms of elderberry supplements compared with no supplements, placebo, or other active interventions for preventing viral respiratory infections;
2. To assess the benefits and harms of elderberry supplements compared with no supplements, placebo, or other active interventions for treating viral respiratory infections; and.
3. To assess the relationship between elderberry supplements and negative health impacts associated with the overproduction of pro-inflammatory cytokines.

### Eligibility criteria

Study selection criteria were established in the context of the PICOS (Population – Intervention – Comparators – Outcomes – Study design) framework and are described next.

#### Participants

Studies including participants with the goal of preventing or treating viral respiratory infections were considered for inclusion. Included viral respiratory infections were the common cold, influenza, and infections due to novel coronaviruses (including severe acute respiratory syndrome (SARS), Middle East respiratory syndrome (MERS), and COVID-19). Studies of prevention were required to include people not yet diagnosed with the common cold, influenza, or an infection due to a novel coronavirus, and studies of treatment had to include people diagnosed (by any criterion) with the common cold, influenza, or a novel coronavirus infection. We grouped studies on different infections (e.g., the common cold, influenza) separately for all analyses. Studies on bacterial respiratory infections were excluded from this systematic review. Further, studies of treatment for other virally triggered respiratory infections (e.g. rhinosinusitis), were also excluded, other than when characterized as a symptom of the common cold, influenza or novel coronavirus infections. There were no exclusions based on participant age, gender, comorbidities or setting.

To capture additional evidence on the relationship between elderberry and cytokine production, we also included studies with participants who were given elderberry, for any reason, and for whom cytokine production was measured after provision of elderberry. Our rationale for expanding the population for this question was that effects on cytokines might not be dependent on the indication for use, and we wished to capture all available information on this potentially serious outcome.

#### Interventions

Although the elderberry species most commonly used for immune support is black elderberry (*Sambucus nigra*; also known as European elderberry), other species of Sambucus with similar characteristics (e.g., *Sambucus ebulus*, *Sambucus canadensis*) are sometimes used. We therefore included interventions using any elderberry species. There were no exclusions based on elderberry dose, frequency, preparation method, or mode of delivery, and no minimum duration of intervention. Because elderberry is sometimes taken in combination with other herbal products (e.g., echinacea spp.), we included studies assessing elderberry products in which elderberry was one of the components of the herbal intervention. However, we grouped elderberry-only studies and studies in which the intervention was elderberry in combination with other products separately in all analyses.

#### Comparators

We included studies comparing elderberry to: 1) no supplementation, 2) placebo, 3) a different formulation, dose, or schedule of elderberry, or 4) a non-elderberry active control (e.g., vitamin D). Our rationale for comparisons between different elderberry interventions was to detect whether particular formulations, doses or schedules of elderberry are different in effectiveness. We also included studies in which elderberry was taken as an adjunct to another intervention and compared this use to 1) no adjunctive intervention, 2) adjunctive placebo, or 3) another active adjunctive intervention, including both conventional and complementary interventions.

#### Outcomes

For the objective of assessing the ability of elderberry to prevent viral respiratory infection, we included the following outcomes: 1) Number of new cases of infection, 2) severity of illness (as defined in the studies) among new cases, 3) total duration of illness, and 4) adverse events / harms as defined and reported in the studies.

For the objective of assessing the ability of elderberry to treat viral respiratory infection, we included the following outcomes: 1) time to improvement in viral illness, 2) total duration of viral illness, 3) incidence of hospitalizations, 4) duration of hospitalization, 5) frequency of intubation and ventilation, 6) mortality, and 7) adverse events / harms as defined and reported in the studies.

For the objective of assessing the relationship between elderberry supplements and negative health impacts associated with the overproduction of pro-inflammatory cytokines, we included the following outcomes: 1) cases of systemic sepsis, 2) cases of multi-organ failure, and 3) expression of cytokines, including interferons (IFNs), interleukins (ILs), chemokines, colony-stimulating factors (CSFs), c-reactive protein (CRP) and tumor necrosis factor (TNF-alpha) in vivo, preferentially measured as changes in concentration from baseline.

We did not have any restrictions on duration of follow-up for any of the included outcomes.

#### Study designs

For the objectives of assessing the effects of elderberry in preventing or treating viral respiratory infections, we included randomized controlled trials (RCTs) only. The rationale for this was that properly conducted RCTs are the least biased sources of evidence on the effectiveness of an intervention. For the objective of assessing the relationship between elderberry supplements and negative health impacts associated with the overproduction of pro-inflammatory cytokines, we included any available information from RCTs. However, we also sought evidence from cohort studies, controlled before-and-after studies, interrupted time series, case-control studies, and case reports. We also included studies that looked directly at cytokine production, even if clinical events were not measured. The rationale for this was to be maximally inclusive of evidence that may inform any relationship between elderberry and risk of so-called ‘cytokine storm’. In all studies looking at elderberry and inflammation, we sought to capture factors that may confound the association between elderberry and cytokine production or risk of systemic sepsis or multi-organ failure, paying particular attention to autoimmune conditions and other pre-existing immunity characteristics.

### Literature search and screening

We searched six databases (MEDLINE (PubMed), CENTRAL, EMBASE, CABI, Science Citation Index, and International Pharmaceutical Abstracts), four research registers (WHO COVID-19 Global Research Database, LIT-COVID, Center for Disease Control and Prevention COVID-10 Research Article Database, and Clinicaltrials.gov), and two preprint sites (MedRixv, BioRxiv) on June 11, 2020 without date restrictions. We used a search strategy developed by an information specialist (EL), peer-reviewed by another information specialist [19], and modified for each source. See Additional file 1 for the MEDLINE (PubMed) search strategy and peer review form. We also checked the reference lists of related systematic reviews and the reference lists of all included studies. We did not exclude studies on the basis of language or publication status.

We imported all references directly into to Covidence, where they were deduplicated [20]. Two authors (LSW, VP) independently screened the titles and abstracts and all records identified as potentially relevant by either author were obtained in full text. Two authors (LSW and VP, SK, or DS) independently reviewed each of the full texts and decided upon study inclusion or exclusion. Disagreements were resolved by discussion or involvement of a third author.

### Data extraction and risk of Bias appraisal

One author (LSW) extracted study characteristics (including country and setting, participant characteristics, intervention characteristics, outcomes assessed) into Covidence and a second author (VP) checked the data. Two authors (LSW, VP) then independently extracted numerical data for the outcomes into a Microsoft® Excel spreadsheet and any disagreements were resolved by discussion. Measures of treatment effect and the total number of participants in both the treatment and control groups were recorded. If point estimates or measures of dispersion were not available, we made every effort to estimate as accurately as possible using the provided data. Where necessary, data were read from graphs. Two authors (LSW, VP) used the Cochrane Risk of Bias 1.0 criteria [21] to independently assess the risk of bias for each included RCT. The same authors used the Cochrane Effective Practice and Organisation of Care (EPOC) criteria [22] to assess the risk of bias for controlled before-and-after (CBA) and interrupted time series (ITS) studies. Any disagreements between authors were resolved by discussion.

### Evidence syntheses and appraisals of the strength of evidence

For RCTs of treatment or prevention in which the participants, interventions, comparators and outcomes were similar, we carried out meta-analyses in RevMan 5.4 [23]. We combined data across studies using a random effects model because we expected the individual studies to have clinical and methodological heterogeneity and wished to generalise the findings to broadly similar studies. When data were available for outcomes but were not appropriate for pooling for reasons related to clinical or methodologic heterogeneity, we presented the information in forest plots without meta-analyses. We used risk ratios (RR) for dichotomous data and mean differences (MD) for continuous data, and 95% confidence intervals (CI) for all effect estimates were also estimated. We used visual inspection of forest plots, statistical tests (chi-square test with p < =0.1), and the I^2^ statistic to evaluate the presence and extent of heterogeneity [24]. We planned to conduct subgroup analyses by types of viral illness (e.g., influenza versus the common cold), study population demographics (e.g., adults versus children) clinical characteristics (e.g., baseline severity of illness, vaccination status), and elderberry characteristics (e.g., dose, delivery method), to understand potential sources of heterogeneity if sufficient data were available. We also planned to conduct sensitivity analyses in which we excluded studies at a high risk of bias for selection, outcome assessment, or loss to follow-up. Finally, we planned to assess reporting biases with funnel plots if there were at least 10 trials in a comparison, however no analysis included more than two trials*.*

For studies related to ‘cytokine storm’ and other outcomes such as cytokine production, we did not plan to statistically pool data across studies because we expected the study questions to vary and study designs to include controlled and uncontrolled experiments and observational studies, rendering the overall body of evidence insufficiently similar for statistical pooling. Instead, we carried out a narrative synthesis of the evidence from the included studies.

We used the Grading of Recommendations Assessment, Development and Evaluation (GRADE) framework to evaluate the certainty of the evidence for all pre-planned outcomes related to prevention and treatment [25]. Two authors (VP, LSW) independently extracted the individual and summary GRADE assessments into a Word table and resolved any disagreements by discussion. We used the Preferred Reporting Items for Systematic reviews and Meta-Analyses (PRISMA) to guide the reporting of this systematic review [26].

## Results

### Extent of available literature

We retrieved a total of 1187 records from the searches of databasess, preprint servers and research registers. After screening of the titles, abstracts, and full texts was completed, eight studies were included in this review. A total of 69 studies were excluded during full text screening, primarily because they were in-vitro studies. We identified five studies that may have been completed but do not yet have publications [27–31], and one study that is ongoing [32]. We did not identify any completed studies from preprint sources or otherwise outside the peer-reviewed literature. Additional file 2 provides a listing of excluded studies and reasons for exclusion.

For the objective of assessing the effects of elderberry in the prevention of viral respiratory illness, we found one RCT [33] with 312 participants evaluating elderberry compared to placebo for the prevention of symptoms of the common cold. For the objective of assessing the effects of elderberry in the treatment of viral respiratory illness, we found three RCTs [10, 34, 35] with 151 participants evaluating elderberry compared to placebo for the treatment of influenza and one RCT [36] with 473 participants evaluating a product containing echinacea and elderberry (Echinaforce® Hotdrink) compared to oseltamivir for the treatment of influenza. Because our original searches were focused on elderberry and we may have missed additional studies on this mixed product, we repeated our database searches in March 2020 with the text word ‘Echinaforce’. The search retrieved 79 references, however with the exception of the Echinaforce® Hotdrink trial registration (EudraCT Number 2010–021571-88) all retrieved references potentially related to randomized controlled trials were to forms of Echinaforce® that do not contain elderberry (e.g., Echinaforce® drops, Echinaforce® Forte) and we excluded them at title and abstract stage. Table 1 provides a summary of the characteristics of each included study, while Additional file 3 presents a comprehensive description of study characteristics, including all available information on the constituents and standardization of the elderberry products.

**Table 1:** Characteristics of prevention and treatment studies

Study	Country/ date/ setting	Participants analyzed; age; gender; baseline symptoms	Duration of study	Intervention	Control	Outcomes Measured	Safety Evaluation	Sponsor
**Prevention of the common cold with elderberry**								
Tiralongo 2016 [33]	Australia/ 2013–14/ outpatient	*N* = 312; mean 51 (sd 16) years; 106 (34%) men; participants were in good general health and planning to travel overseas	15–16 days	Capsule with 300 mg proprietary elderberry extract taken 2–3 times/day	Placebo capsule	Proportion developing a cold, number of cold episode days, assessment of eight specific cold symptoms on a 0–4 scale (Jackson scale), quality of life, use of concomitant medication or therapy.	Although some minor adverse events were mentioned, assessment was not described.	Iprona AG, Italy.
**Treatment of influenza with elderberry**								
Kong 2009 [35]	China/ 2009/ outpatient	*N* = 64; mean 40 years (range 20–59); 34 (53%) men; symptoms of influenza for <24 h	2 days	Lozenge with 175 mg proprietary elderberry extract taken 4 times/day	Placebo lozenge	Percentage improving from baseline symptoms of headache, nasal congestion, muscle aches, coughing, mucus discharge or fever, and mean VAS status for each symptom.	No mention of specific ascertainment however lack of side effects in the elderberry group was mentioned.	HerbalScience Singapore Pte. Ltd.
Zakay-Rones 2004 [10]	Israel/ 1999–2000/ outpatient	N = 60; mean 30 (2.9) years; 33 (55%) men; symptoms of influenza for a mean of 27 h	5 days	15 ml syrup with 38% proprietary elderberry extract (Sambucol) taken 4 times/day	Placebo syrup	Mean VAS status for global well-being and for six symptoms.	Participants were asked if they had any problem with sedation.	Razei Bar, Jerusalem, Israel
Zakay-Rones 1995 [34]	Israel/ not reported/ outpatient	N = 27; range 5–56 years; 18 (67%) men; symptoms of influenza for <24 h	3 days	1 tablespoon syrup with proprietary elderberry extract (Sambucol) taken 2 times/day (children) or 4 times/day (adults)	Placebo syrup	Proportion with complete cure and with improvement in specific symptoms. Overall duration of illness and mean number of days for seven symptoms.	Prior to study 35 persons received 4 tablespoons Sambucol daily for 3 days and no side effects were recorded.	Not reported
Treatment of influenza with elderberry and echinacea								
Rauš 2015 [36]	Czech Republic/ 2011–13/ outpatient	*N* = 420; mean 37.2 (sd 13.5) years; 210 (50%) men; symptoms of influenza for ≤48 h	10 days	5 ml Echinaforce Hotdrink (combines echinacea and elderberry) taken 3–5 times/day (+ oseltamivir placebo taken 2 times/day)	Tamiflu taken 2 times/day (+ 5 ml Echinaforce Hotdrink placebo taken 3–5 times/day)	Proportion recovered from symptoms, proportion with complications, severity of nine symptoms, sleep disturbance, return to normal activity, body temperature, use of rescue medication, health care contacts, need for antibiotics, hospitalization, and tolerability	Participants were asked whether they experienced any unusual or unexpected symptoms in addition to their influenza symptoms.	A. Vogel Bioforce AG, Roggwil, Switzerland

For the objective of assessing the relationship between elderberry and risk of systemic sepsis or multi-organ failure, we did not locate any studies, of any design, in which these clinical outcomes were measured. However, we identified three studies in which a total of 51 people were given elderberry products and cytokine production was subsequently assessed (ex vivo studies). One of these studies was an RCT [37] comparing 12 weeks of either elderberry or placebo in 52 healthy post-menopausal women, one study was a single-arm cohort study [38] in which 22 healthy volunteers drank *Sambucus ebulus* (i.e. European dwarf elderberry) tea for 30 days, and one study evaluated cytokines before and after single oral doses of both diclofenac and elderberry [15, 39]. The diclofenac and elderberry study appeared in two publications with the same first author and the same outcome means and standard deviations at each time point; we contacted the first author for clarification and did not receive a response so for this review we considered the publications to report the same study. In all included ex vivo studies, various cytokines were measured at baseline and then at one or more time points after the use of elderberry. Table 2 provides a summary of the characteristics of each included study.

**Table 2:** Characteristics and results of ex vivo cytokine studies

Study	Study design, participants and methods	Cytokines	Results
Kirichenko 2016 [15, 39]	Comparative before and after study Participants were healthy volunteers but number of participants is not clear. Participants received elderberry tincture and diclofenac. Cytokines were measured before and 2, 4, and 8 h after oral tincture of elderberry and also before and 2, 4, and 8 h after oral diclofenac.	IL-1 and TNF-alpha	After elderberry, reductions from baseline in IL-1 were seen at 2, 4, and 8 h after intake and reductions from baseline in TNF-alpha were seen at 2 h. After diclofenac, reductions from baseline in both IL-1 and TNF-alpha were statistically significant at 2, 4, and 8 h. With elderberry the mean percentage ± SEM from baseline for IL-1 was 68.0 ± 2.6 at 2 h, 70.7 ± 2.4 at 4 h, and 88.3 ± 2.0 at 8 h. The decrease was statistically significant (*p* < 0.05) at each point. TNF-alpha levels were 80.3 ± 2.0 at 2 h, 98.0 ± 14.1 at 4 h, and 121.7 ± 21.6 at 8 h. The decrease was significant (p < 0.05) only at 2 h. With diclofenac, IL-1 levels were 78.0 ± 5.5 at 2 h, 73.3 ± 7.9 at 4 h, and 51.0 ± 21.8 at 8 h and TNF-alpha levels were 80.0 ± 10.0 at 2 h, 88.0 ± 7.5 at 4 h, and 61.3 ± 6.6 at 8 h. The effects of diclofenac were significant (p < 0.05) for both cytokines at each time point.
Ivanova 2015 [38]	Before and after study *N* = 22 healthy volunteers drank elderberry tea for 30 days. Cytokines were measured at baseline and after treatment.	IL-beta, CRP, IL-6, TNF-alpha	After 30 days of elderberry, there were reductions in IL-beta and in CRP but little or no reductions in IL-6 and TNF-alpha. IL-beta (mean ± SEM) (data read from Fig. 2) was 16 ± 3.7 pg/mL at day 0 and 12 ± 2.2 pg/mL at day 30 (p < 0.05), CRP was 1.25 ± 0.41 mg/L at day 0 and 0.84 ± 0.32 mg/L at day 30 (p < 0.05). IL-6 was 18.33 ± 4.10 pg/mL at baseline and 15.09 ± 3.04 pg/mL at day 30). TNF-alpha was 21.13 ± 10.73 pg/mL at day 0 and 10.79 ± 2.67 pg/mL at day 30)
Curtis 2009 [37]	RCT *N* = 52 healthy postmenopausal women took either elderberry capsules (*n* = 26) or placebo capsules (n = 26) for 12 weeks. Cytokines were measured at baseline and after treatment.	CRP, TNF-alpha, IL-6	After 12 weeks of supplementation with elderberry or placebo, there was no difference in plasma levels of cytokines. The mean ± SD of IL-6 (ng/L) was 1.0 ± 1.4 at baseline and 0.9 ± 0.9 at 12 weeks in placebo group, compared to 1.0 ± 0.9 at baseline and 1.0 ± 0.6 at 12 weeks in elderberry group. TNF-alpha (ng/L) was 14.8 ± 9.3 at baseline and 13.0 ± 9.2 at 12 weeks in placebo group, compared to 15.3 ± 11.1 at baseline and 10.5 ± 5.5 at 12 weeks in elderberry group. CRP (mg/L) was 0.9 ± 0.9 at baseline and 0.9 ± 0.7 at 12 weeks in placebo group, compared to 1.3 ± 1.0 at baseline and 1.3 ± 1.1 at 12 weeks in elderberry group

**Fig. 1:**
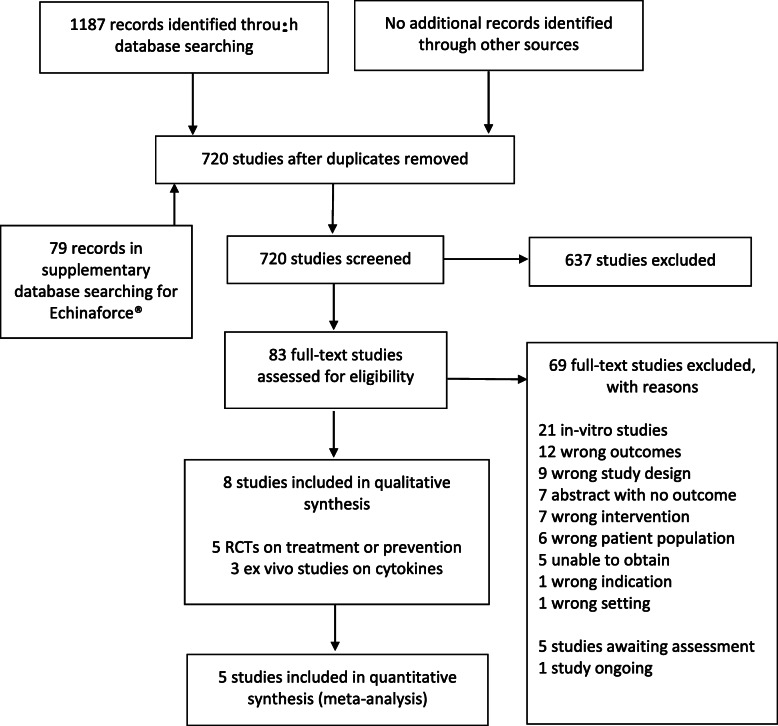
Flow of records through the study selection process

Figure 1 presents a summary of the study search and selection process [26].

### Risk of Bias and certainty of the evidence

**Fig. 2:**
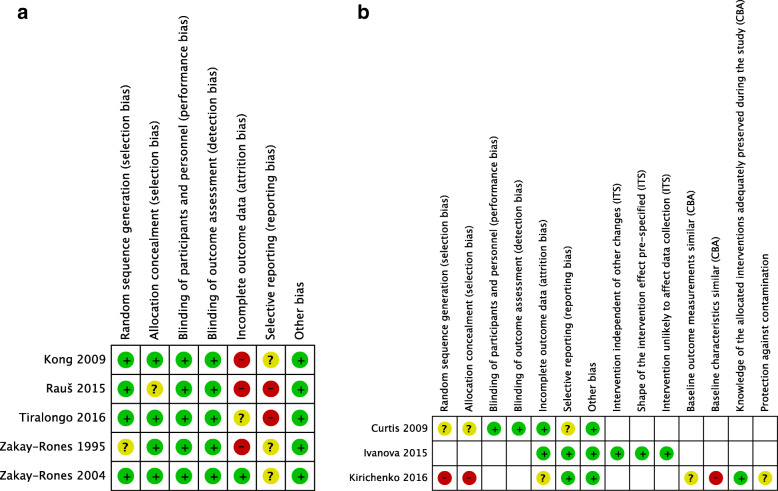
**a**: Risk of bias for randomized trials of prevention or treatment. **b**. Risk of bias for ex vivo studies of elderberry and cytokines

Figure 2a presents the risk of bias judgements for each randomized trial of prevention or treatment. We rated Zakay-Rones 1995 at unclear risk of bias for random sequence generation because the randomization process was not described, and Raus 2015 at unclear risk of bias from allocation concealment because the allocation process was not described. There was a high risk of bias from incomplete outcome assessment due to dropouts, per-protocol analyses or lack of clarity about the numbers of participants in Kong, Raus, Tiralongo, and Zakay-Rones 1995. Neither protocols nor registrations were available for Kong, Raus, Zakay-Rones 1995, or Zakay-Rones 2004, and we considered the risk of bias from selective outcome reporting to be unclear except in Raus 2015, in which some outcomes described in the methods were missing or unclear from the results; we therefore judged the risk of bias as high. A trial registration was available for Tiralongo, however the primary outcome in the trial registration of symptom days did not correspond to the primary reported outcome of defined cold episode days, and we therefore rated the risk of bias from selective outcome reporting as high. We rated the remaining domains of prevention and treatment studies [10, 33–36] at low risk of bias.

Figure 2b provides a representation of the risk of bias judgements for each ex vivo study. We used the Cochrane risk of bias tool to assess the randomized trial assessing cytokines ex vivo after elderberry versus placebo (Curtis 2009) [37]. The report did not include any information on randomization and allocation procedures, and we rated it at unclear risk of bias for sequence generation and allocation concealment. Because the trial was double-blinded and assessed objective outcomes, we rated it at low risk of bias from blinding of participants, personnel, and outcome assessors. Due to no available protocol we rated the report at unclear risk of bias from selective outcome reporting. There was no other apparent risk of bias. We considered the interrupted time series rating to be the most appropriate EPOC tool for rating the risk of bias for the single-arm cohort study assessing cytokines before and after 30 days of elderberry tea (Ivanova 2015) [38] and we rated the study as low risk of bias in all domains. We considered the controlled before and after rating to be the most appropriate EPOC tool for rating the risk of bias for the study comparing cytokines before and after elderberry and diclofenac (Kirichenko 2016) [15, 39]. The study reports were unclear about the characteristics of participants and the study methods. For example, it was unclear whether the diclofenac and elderberry were provided to the same or different participants, and if the former, descriptions regarding a defined order of interventions and a washout period were lacking. We rated the study at high risk of bias for sequence generation and allocation concealment (as is required for controlled before and after studies) but also for no information on the baseline characteristics. We also rated the study at unclear risk of bias for baseline outcome measurements, incomplete outcome data, and protection against contamination.

We evaluated the GRADE level of evidence on the effects of elderberry upon the prevention and treatment of viral respiratory illness as moderate to very low certainty, primarily due to problems with the conduct of the studies and imprecision in the effect estimate as a result of small sample sizes or numbers of events. Additional file 4 details the GRADE assessments of the certainty of the evidence for each outcome reported in this manuscript.

### Elderberry and prevention of respiratory illness

**Fig. 3:**
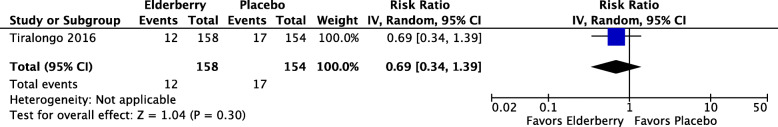
Number of participants developing a cold

Among the 312 adult participants completing assessment in the only RCT evaluating elderberry extract for the prevention of symptoms of the common cold (Tiralongo 2016), 12/154 (8%) of those in the elderberry group and 17/158 (11%) in the placebo group developed a well-defined cold (RR 0.69, 95% CI 0.34 to 1.39; *p* = 0.30) (see Fig. 3). We rated the certainty of this evidence as low, due to risk of bias and imprecision. There were 2 adverse events in the placebo group and 3 adverse events in the elderberry group (RR 0.65, 95% CI 0.11 to 3.84; *p* = 0.63) and because of the very small number of events and the width of the confidence interval, we assessed the certainty of this estimate as very low.

**Fig. 4:**
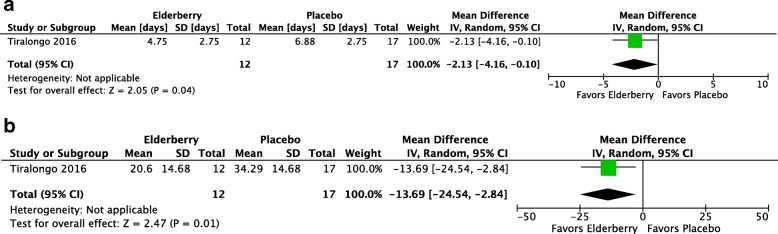
**a**. Mean duration of colds (days) among persons developing colds. **b**. Mean severity of colds (Jackson score) among persons developing colds

The Tiralongo study defined the presence and severity of the common cold using the Jackson Score. Participants recorded daily whether they believed they had a cold and assessed the severity of eight cold symptoms on a 4-point Likert Scale (0 = no symptoms, 3 = severe symptoms). A well-defined cold episode was present when participants believed they had a cold and also had a minimum total symptom score of 14, summed over at least six consecutive days. Among the 29 participants developing a cold in this trial, the mean duration of the cold was 2 days shorter in the elderberry group (MD -2.13, 95% -4.16 to − 0.10; *p* = 0.04, see Fig. 4a) and the mean symptom severity of the cold was lower (MD -13.69, 95% CI − 24.54 to − 2.84; *p* = 0.01, see Fig. 4b). Due to risk of bias concerns and imprecision, we assessed the certainty of both estimates as very low.

### Elderberry and treatment of respiratory illness

**Fig. 5:**
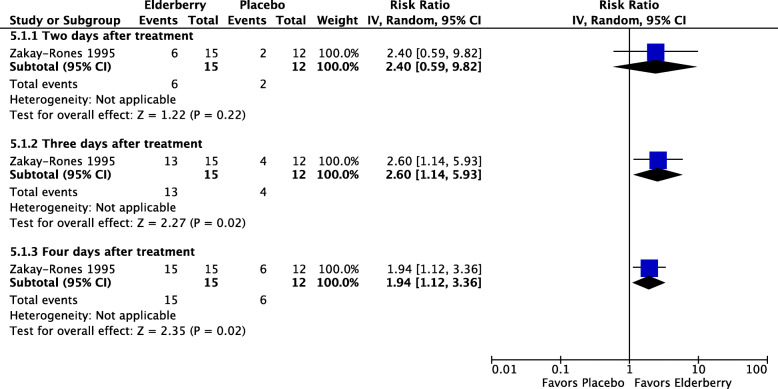
Number of participants recovering from influenza

**Fig. 6:**
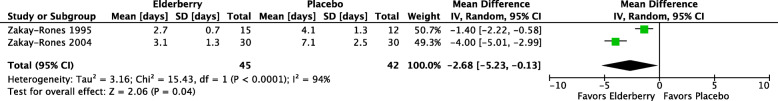
Time to resolution or global improvement (days)

Three studies [10, 34, 35] evaluated proprietary elderberry products for the treatment of influenza symptoms. Two studies (Zakay-Rones 1995, Zakay-Rones 2004) tested Sambucol® versus placebo and one later study (Kong 2009) tested a different proprietary elderberry product (not named in the study report) versus placebo. The two earlier studies analyzed a total of 87 participants, including both children and adults, with symptoms of influenza A or influenza B. Zakay-Rones 1995 (*n* = 27) measured the mean duration of illness and Zakay-Rones 2004 (*n* = 60) measured time to improvement in each influenza symptom as well as time to global improvement on a visual analogue scale (VAS) (0 to 10; 0 = no problems, 10 = pronounced problems) and time to complete cure. Compared to participants taking placebo, participants taking elderberry had higher rates of cure at two days (RR 2.40; 95% CI 0.59 to 9.82), three days (RR 2.60, 95% CI 1.14 to 5.93), and four days (RR 1.94, 95% CI 1.12 to 3.36) after beginning treatment (see Fig. 5). The overall time to resolution or global improvement of illness was nearly three days shorter in the elderberry group compared to the placebo group (MD − 2.68 days, 95% CI − 5.23 to − 0.13; 2 studies, 87 participants; I^2^ = 94%) (see Fig. 6). However, the certainty of these estimates is very low due to risk of bias concerns, the involvement of a small number of participants, and, with respect to the overall time to global improvement or cure, inconsistency between studies (I^2^ = 94%). The later study (Kong 2009) analyzed a total of 64 participants randomized to elderberry or placebo, and measured the duration and severity of individual symptoms, but did not present our prespecified outcomes of overall duration or severity of illness; we were unable to reach the author or calculate this outcome from the available data. (See Additional file 5 for forest plots of reported outcomes from Zakay-Rones 1995 and Kong 2009 that were not prespecified for inclusion in this review.) None of the three studies reported any adverse effects in either the elderberry group or the placebo group, however the definition and procedures for assessment of adverse events was inconsistent between studies and the certainty of this finding is very low.

**Fig. 7:**
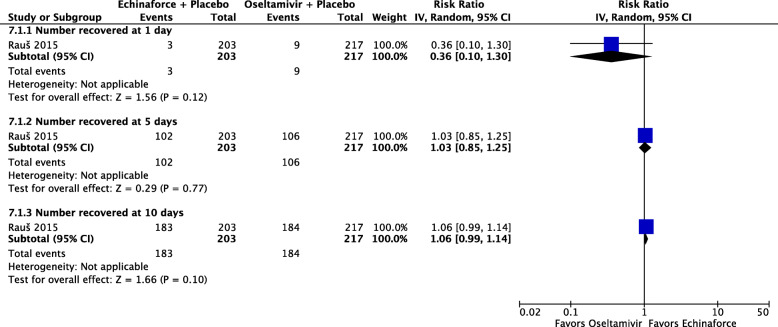
Number of participants recovering from influenza

**Fig. 8:**
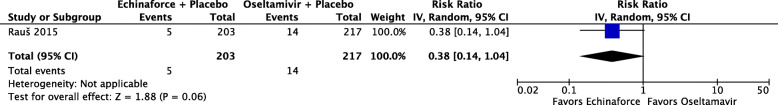
Number of participants developing complications

**Fig. 9:**
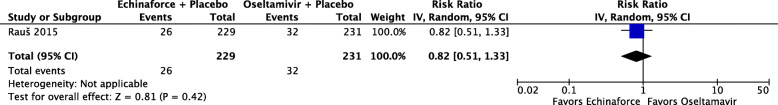
Number of participants with adverse events

One study, reporting on 420 participants [36], compared a combined echinacea and elderberry product (Echinaforce® Hotdrink) plus placebo to oseltamivir (Tamiflu®) plus placebo for treatment of influenza. There is low certainty evidence that fewer people receiving the echinacea/elderberry product may recover after one day compared to people receiving oseltamivir (RR 0.36, 95% CI 0.10 to 1.30), low-certainty evidence of little to no differences in recovery rates at three days (RR 1.03, 95% CI 0.85 to 1.25), and moderate-certainty evidence of no difference at five days (RR 1.06, 95% CI 0.99 to 1.14) (see Fig. 7). People receiving the echinacea/elderberry product may have a lower risk of complications (RR 0.38, 95% CI 0.14 to 1.04; see Fig. 8) and adverse events (RR 0.82, 95% CI 0.51 to 1.33; see Fig. 9) when compared to people taking oseltamivir, but the certainty of evidence is low due to the risk of bias and a small number of events.

We did not find sufficient available data to carry out subgroup analyses by study population demographics, clinical characteristics, or characteristics of the elderberry intervention such as dose or type of elderberry formulation. For sensitivity analyses excluding studies at high risk of bias, only one study (Zakay-Rones 2004) did not have a high risk of bias for selection, outcome assessment, or loss to follow-up, and a sensitivity analysis can be approximated by observing the effect estimates of the two studies in the only meta-analysis (Fig. 6)*.*

### Elderberry and inflammation-related outcomes

We did not quantatively synthesize data across ex vivo studies because of the different study designs. Table 2 provides a summary of the findings from each study. Results of one randomized controlled trial suggested no evidence for a difference in levels of CRP, TNF-alpha, or IL-6 between healthy postmenopausal women who were randomized to 12 weeks of elderberry capsules (a dose of 500 mg/day anthocyanin (as cyanidin-3-glucoside)) or to 12 weeks of placebo [15]. A before-and-after study reported on 22 healthy volunteers who drank a 200 ml elderberry infusion (an approximate dose of 3.66 mg anthocyanins per liter) for 30 days. This study suggests that there is evidence for statistically significantly lower serum CRP and IL-beta levels after 30 days of elderberry intake, but little to no difference in IL-6 and TNF-alpha [38]. Finally, after a single administration of 2.5 g of black elderberry tincture was given to 3 healthy volunteers, there was a statistically significant reduction from baseline in IL-6 levels measured at 2, 4 and 8 h and a statistically significant reduction from baseline in TNF-alpha levels at 2 h, but not at 6 or 8 h [15, 39]. This study also assessed the effect of 100 mg of diclofenac upon cytokines and observed that after diclofenac, both IL-6 and TNF-alpha levels were significantly reduced from baseline at 2, 4, and 8 h.

## Discussion

### Implications of findings

Based on evidence from one large study of healthy adults, elderberry may not reduce the risk of developing the common cold, although it remains possible that colds developed during elderberry supplementation may be shorter and less severe than colds developed on placebo. However, there is insufficient information to be certain about these effects.

Based on three studies testing elderberry versus placebo for its effect on symptoms of influenza, it is possible that illness may be shorter and less severe with elderberry than with placebo. However, the estimates of quicker recovery are very uncertain because the studies to date have been small and are not without problems in conduct (e.g., incomplete outcome data, selective presentation of outcomes) and the estimate of mean difference in days to recovery displays high heterogeneity (I^2^ = 94%). Although no serious adverse events were reported in any study, the limited attention to adverse events overall means that we are very uncertain about types and rates of more minor adverse events. We are aware of two recently completed but not yet published studies testing Sambucol® for cold or influenza symptoms [27, 40] and will incorporate the results of these trials in a future update of this review.

Based on one study comparing a mixed herbal product incorporating elderberry (Echinaforce® Hotdrink) to oseltamivir, there may be a slightly higher rate of recovery with oseltamivir at one day after beginning treatment, but little or no difference between treatments in rates of recovery at five and ten days. There may be a lower risk of complications or adverse events with the herbal preparation compared to oseltamivir, and it appears to be a viable option for treatment. We are aware of recently completed or ongoing studies comparing mixed elderberry products to placebo for respiratory symptoms [29, 41]. Information from these studies will further clarify the potential role of these mixed products containing elderberry in respiratory illness. Overall, further research is needed to establish whether elderberry (in different forms and at different doses) is effective in either preventing or ameliorating respiratory illnesses (including not only colds and influenza but also illnesses resulting from novel coronaviruses) in populations of different ages and different baseline health statuses.

We did not find any evidence on the impact of elderberry on clinically relevant outcomes related to inflammation, however, we found three studies examining ex vivo effects of elderberry in healthy adults. We expected to see reductions in cytokine levels ex vivo consistent with findings from in vitro studies [14, 42]. While there were some statistically significant reductions in cytokines indicating that elderberry likely has some effect on inflammatory markers, the evidence was underwhelming in the studies with interventions of longer duration, suggesting that this effect may abate over time with repeated dosing. The comparison to diclofenac in the small study using single doses of elderberry or diclofenac, however, does provide some context for the reader as diclofenac is recognized as a potent non-steroidal anti-inflammatory drug, and the study suggests that elderberry is as effective or less effective than diclofenac in IL-1 reduction over time [15]. Based on the ex vivo evidence, there does not currently appear to be any reason for concern about elderberry products and risk of overstimulation of the immune system. However, in order to determine the clinical significance of elderberry’s effect on inflammation and cytokine storm, future trials must involve patients with inflammatory conditions and evaluate more meaningful clinical outcomes associated with inflammation in addition to surrogate markers such as cytokine serum concentrations.

We are aware of one previous systematic review of elderberry for viral respiratory illness (Hawkins 2019) [8]. As mentioned in the Introduction, that review examined elderberry only for the treatment of respiratory illness. It did not look at the prevention of illness, the use of mixed herbal elderberry products, or the effects of elderberry upon clinical or ex vivo cytokine outcomes. Our assessment of the effects of elderberry for treatment of viral respiratory illnesses do not conflict with the findings of the previous review, in that we also observed benefits. However, we believe that our review provides a more accurate assessment of the quality of the available studies and the certainty of the findings. The previous review used the 27-item Downs and Black checklist to rate the studies and found that the overall risk of bias in the studies was low. We caution that the use of quality checklists may not adequately reflect risk of bias [43]. We chose to use the Cochrane risk of bias tool and identified problems with the conduct of each of the studies that raised some concerns. We then incorporated this assessment of risk of bias, together with imprecision due to the very small numbers of study participants, into GRADE judgements of low or very low certainty for all estimates of the effects of elderberry versus placebo in treating respiratory illness. We expect that information from future studies may revise these estimates and provide more reliable evidence.

### Limitations

This review is limited by the small number, low quality, and limited information on subgroup factors available from included studies. The conduct of the review itself has both strengths and limitations. Although the review was originally conceived as a ‘rapid’ review, which implies the use of some shortcuts in methods to improve speed, in the end we did not abbreviate any systematic review methods (e.g., we performed dual screening of all titles and abstracts). One limitation of our review was that, unlike the previous systematic review (Hawkins 2019), we were unable to make contact with authors or calculate an acceptable overall duration or severity of illness for the Kong 2009 treatment study. Although we were not able to obtain data for these outcomes and incorporate them into meta-analyses, we believe that the relatively small sample size and high risk of bias for Kong 2009 would not have improved the GRADE level of evidence for either duration or severity of illness. Therefore, while our conclusions are consistent with those of the earlier review, we believe we are appropriately conservative about the certainty of the evidence, and we await the results of ongoing trials for a more conclusive picture.

While our review was comprehensive in identifying products containing solely elderberry it has limitations in the identification of products mixing elderberry and other constitutents (e.g., echinacea). Once we had identified the Echinaforce® Hotdrink trial, we carried out a ‘top up’ search to check for any additional studies of Echinaforce® and we can be confident that we did not miss additional studies of Echinaforce® plus elderberry. Because our primary goal was to assess elderberry products, we did not search for other herbs or herbal products, and although we identified the Echinaforce® Hotdrink trial and two ongoing trials of mixed elderberry products [29, 41] we might have found additional trials of mixed products with more extensive searching. This review does not claim to be a comprehensive look at mixed elderberry products, which might be the subject of further research.

### Changes between protocol and review

We made some clarifications and changes to the exclusion criteria and outcomes outlined in the protocol. For example, we specified that we would include studies of the common cold, influenza, or infections with novel coronaviruses, but we did not explicitly state that we would exclude studies on other infections that could potentially originate from these viruses. It was decided post hoc to exclude studies on respiratory infections (e.g., acute rhinosinusitis) that were not explicitly linked to the common cold, influenza, or coronavirus because acute rhinosinusitis could be caused by bacterial or fungal infections rather than viral infections. Likewise, we specified that we would include studies in which people were given elderberry for any reason and production of cytokines was measured afterwards, but we did not explicitly exclude studies conducted entirely in vitro, and we only made this decision post hoc.

For treatment studies, we planned to extract information on time to improvement in symptoms of viral illness, but we replaced this outcome with time to improvement in illness, as we believe this to be a more relevant outcome than duration of individual symptoms. Finally, for prevention studies, we originally planned to report the number of persons newly experiencing specific upper respiratory disease symptoms, but we replaced this outcome with total duration of illness because we felt that overall illness was a more salient outcome than the duration of individual symptoms.

## Conclusions

Elderberry is a promising intervention for reducing the severity and duration of influenza and the common cold, and it does not appear associated with serious adverse effects. However, the current evidence base is limited in both size and quality. The results of ongoing and recently completed but not yet published studies may provide more conclusive evidence on potential benefits and harms and allow exploration of subgroup factors. In the meantime, we have not identified any cause for concern about the overstimulation of the immune system during elderberry supplementation. However, additional searches for clinically relevant reports should be repeated in the future.

## Supplementary Information

**Additional file 1.**

**Additional file 2.**

**Additional file 3.**

**Additional file 4.**

**Additional file 5.**

## Data Availability

All data are extracted from publicly available literature. The data used to support the findings of this study are available from the corresponding author upon request.

## Abbreviations

CBA: Controlled before-and-after
CI: Confidence interval
COVID-19: Coronavirus disease 2019
CRP: C-reactive protein
CSF: colony-stimulating factor
EPOC: Cochrane Effective Practice and Organisation of Care
FDA: U.S. Federal Drug Administration
GRADE: Grading of Recommendations Assessment, Development and Evaluation
IFN: Interferon
IL: Interleukin
ITS: Interrupted-time-series
MD: Mean difference
MERS: Middle East respiratory syndrome
PICOS: Population – Intervention – Comparators – Outcomes – Study design
PRISMA: Preferred Reporting Items for Systematic reviews and Meta-Analyses
RCT: Randomized controlled trial
RR: Risk ratio
SARS: Severe acute respiratory syndrome
SARS-CoV-2: Severe acute respiratory syndrome coronavirus 2
TNF-alpha: Tumor necrosis factor
