# Supplementary material for: Elderberry for prevention and treatment of viral respiratory illnesses: a systematic review
Source: BMC Complement Med Ther. 2021 Apr 7;21:112. doi: 10.1186/s12906-021-03283-5 (PMC8026097; doi:10.1186/s12906-021-03283-5)
Supplement: Supplementary file 1 — Additional file 1. [file 12906_2021_3283_MOESM1_ESM.docx]

Search strategy for MEDLINE (PubMed)

| *(elderberry[tiab] OR elderberries[tiab]OR elderflower*[tiab] OR elder flower*[tiab]OR sambucus[tiab] OR Sambuci flos[tiab] OR s nigra[tiab] OR black elder[tiab] OR “Sambucus”[mesh] OR “Sambucus nigra”[mesh]) AND*  *(influenza*[tiab] OR flu[tiab] OR H1N1[tiab] OR PH1N1[tiab] OR H3N2[tiab] OR AH1N1[tiab] OR A/H1N1[tiab] OR AH3N2[tiab] OR H5N1[tiab] OR common cold[tiab] OR cold virus*[tiab] OR colds[tiab] OR respiratory[tiab] OR LRTI*[tiab] OR RTI[tiab] OR RTis[tiab] OR coronavirus[tiab] OR coronaviruses[tiab] OR corona virus[tiab] OR corona viruses[tiab] OR SARS[tiab] OR MERS[tiab] OR**MERS-CoV[tiab] OR COVID-19[tiab] OR SARS-CoV-2[tiab] OR 2019-nCoV[tiab] OR n-CoV[tiab] OR shock[tiab] OR sepsis[tiab] OR septic*[tiab] OR bacteremia*[tiab] OR bacteraemia*[tiab] OR fungemia*[tiab] OR fungaemia*[tiab] OR parasitemia*[tiab] OR parasitaemia*[tiab] OR viremia*[tiab] OR viraemia*[tiab] OR cytokine*[tiab] OR interleukins[tiab] OR interferons[tiab] OR chemokines[tiab] OR colony-stimulating factor[tiab] OR colony-stimulating factors[tiab] OR tumor necrosis factor[tiab] OR TNF-alpha[tiab] OR “Influenza, Human”[mesh] OR “Influenzavirus A”[mesh] OR “Influenza A Virus”[mesh] OR “Influenza B Virus”[mesh] OR “Common Cold”[mesh] OR “Coronavirus”[mesh] OR “Coronavirus Infections”[mesh] OR “SARS Virus”[mesh] OR “Severe Acute Respiratory Syndrome”[mesh] OR "Middle East Respiratory Syndrome Coronavirus"[Mesh] OR "COVID-19" [Supplementary Concept] OR “Respiratory Tract Infections”[mesh] OR “Shock”[mesh] OR “Shock, Septic”[mesh] OR “Sepsis”[mesh] OR “Cytokines”[mesh] OR “Interleukins”[mesh] OR “Interferons”[mesh] OR “Chemokines”[mesh] OR “Colony-Stimulating Factors”[mesh] OR “Tumor Necrosis Factors”[mesh])* |
| --- |

# *PRESS Guideline* 2015— Search Submission & Peer Review Assessment

Reference: McGowan J, Sampson M, Salzwedel DM, Cogo E, Foerster V, Lefebvre C. PRESS Peer Review of Electronic Search Strategies: 2015 guideline statement. *J Clin Epidemiol* 2016;75:40-6. Available: <http://www.jclinepi.com/article/S0895-4356(16)00058-5/pdf>.

**Search submission: This section to be filled in by the searcher**

Searcher: Emilie Ludeman, MSLIS Email: eludeman@hshsl.umaryland.edu

Date submitted: 6/4/2020 Date requested by: 6/8/2020

| 1. **Systematic Review Title** |  |
| --- | --- |

Elderberry for prevention and treatment of viral respiratory illnesses

| 1. **This search strategy is …** |
| --- |

|  | My PRIMARY (core) database strategy — First time submitting a strategy for search question and database |
| --- | --- |
| X | My PRIMARY (core) strategy — Follow-up review NOT the first time submitting a strategy for search question and database. If this is a response to peer review, itemize the changes made to the review suggestions |
|  | SECONDARY search strategy— First time submitting a strategy for search question and database |
|  | SECONDARY search strategy — NOT the first time submitting a strategy for search question and database. If  this is a response to peer review, itemize the changes made to the review suggestions |

| 1. **Database** (e.g., MEDLINE, CINAHL) *[mandatory]* |
| --- |

MEDLINE

| 1. **Interface** (e.g., Ovid, EbscoHost…) *[mandatory]* |
| --- |

PubMed

| 1. **Research Question** (Describe the purpose of the search)  *[mandatory]* |
| --- |

To assess the benefits and harms of elderberry supplements compared with no supplements, placebo, or other active interventions for preventing or treating viral respiratory infections. To assess the relationship between elderberry supplements and negative health impacts associated with the overproduction of pro-inflammatory cytokines.

| 1. **PICO Format** Outline the PICOs for your question — i.e., Patient, Intervention, Comparison, Outcome, and Study Design — as applicable |
| --- |

| **P** | *Studies including participants with the goal of either preventing or treating participant viral respiratory infections will be considered for inclusion.*  *The included viral respiratory infections will be the common cold, influenza, and infections due to novel coronaviruses (including SARS, MERS, and COVID-19).* |
| --- | --- |
| **I / Exposure** | *Although people often work with black elderberry (Sambucus nigra; also known as European elderberry) for immune support, other species of Sambucus with similar characteristics might also be used, and we will not exclude elderberry interventions based on species.* |
| **C** | *We will include studies comparing elderberry to*  *1) no supplementation,*  *2) placebo, or*  *3) another active control (eg, vitamin D), in which each active control is considered separately.* |
| **O** | *1. For the objective of preventing viral respiratory infection:*  *a) Number of new cases of infection (operationalized in order of priority by self-report, clinical diagnosis, or laboratory test) of viral illness*  *b) Severity of illness (as defined in the studies) among new cases*  *c) Number of persons newly experiencing specific upper respiratory disease symptoms (as defined and reported in the studies)*  *d) Adverse events / harms as defined and reported in the studies*  *2. For the objective of treating viral respiratory infection:*  *a) Time to improvement in symptoms of viral illness*  *b) Total duration of viral illness*  *c) Hospitalization*  *d) Duration of hospitalization*  *e) Frequency of intubation and ventilation*  *f) Mortality*  *g) Adverse events / harms as defined and reported in the studies*  *3. For the objective of assessing the relationship between elderberry supplements and negative health impacts associated with the overproduction of pro-inflammatory cytokines:*  *a) Cases of systemic sepsis*  *b) Cases of multi-organ failure*  *c) Expression of cytokines, including interferons (IFNs), interleukins (ILs), chemokines, colony-stimulating factors (CSFs), and tumor necrosis factor (TNF-alpha) in vivo, preferentially measured as changes in concentration from baseline. Since cytokines vary in sequence of release and concentration over time, all measures will be associated with times after elderberry challenge or times after hospital or ICU admission, as appropriate for the study design and setting.*  *Studies will be included in the review irrespective of whether measured outcome data are reported in a ‘usable’ way.* |
| **S** |  |

| 1. **Inclusion Criteria** (List criteria such as age groups, study designs, etc., to be included) *[optional]*   **This search strategy is …** |
| --- |

| 1. **Exclusion Criteria** (List criteria such as study designs, date limits, etc., to be excluded) **[optional]** |
| --- |

| 1. **Was a search filter applied?** Yes **No** |
| --- |

**If YES, which one(s) (e.g., Cochrane RCT filter, PubMed Clinical Queries filter)? Provide the source if this is a published filter.** *[mandatory if YES to previous question* — *textbox]*

| 1. **Notes or comments you feel would be useful for the peer reviewer**  *[optional]* |
| --- |

| 1. **Please copy and paste your search strategy here, exactly as run, including the number of hits per line. [mandatory]** |
| --- |

(elderberry[tiab] OR elderberries[tiab]OR elderflower*[tiab] OR sambucus[tiab] OR Sambuci flos[tiab] OR s nigra[tiab] OR black elder[tiab] OR “Sambucus”[mesh] OR “Sambucus nigra”[mesh]) AND

(influenza[tiab] OR flu[tiab] OR H1N1[tiab] OR PH1N1[tiab] or H3N2[tiab] or AH1N1[tiab] OR AH3N2[tiab] OR H5N1[tiab] OR common cold[tiab] OR cold virus*[tiab] OR colds[tiab] OR respiratory[tiab] OR coronavirus[tiab] OR coronaviruses[tiab] OR SARS[tiab] OR MERS[tiab] OR MERS-CoV[tiab] OR COVID-19[tiab] OR SARS-CoV-2[tiab] OR 2019-nCoV[tiab] OR n-CoV[tiab] OR shock[tiab] OR sepsis[tiab] OR bacteremia[tiab] OR fungemia[tiab] OR parasitemia[tiab] OR viremia[tiab] OR cytokine*[tiab] OR interleukins[tiab] OR interferons[tiab] OR chemokines[tiab] OR colony-stimulating factor[tiab] OR colony-stimulating factors[tiab] OR tumor necrosis factor[tiab] OR TNF-alpha[tiab] OR “Influenza, Human”[mesh] OR “Influenzavirus A”[mesh] OR “Influenza A Virus”[mesh] OR “Influenza B Virus”[mesh] OR “Common Cold”[mesh] OR “Coronavirus”[mesh] OR “Coronavirus Infections”[mesh] OR “SARS Virus”[mesh] OR “Severe Acute Respiratory Syndrome”[mesh] OR "Middle East Respiratory Syndrome Coronavirus"[Mesh] OR "COVID-19" [Supplementary Concept] OR “Respiratory Tract Infections”[mesh] OR “Shock”[mesh] OR “Shock, Septic”[mesh] OR “Sepsis”[mesh] OR “Cytokines”[mesh] OR “Interleukins”[mesh] OR “Interferons”[mesh] OR “Chemokines”[mesh] OR “Colony-Stimulating Factors”[mesh] OR “Tumor Necrosis Factors”[mesh])

**149 references**

**Peer review assessment: this section to be filled in by the reviewer**

|  | Reviewer: Becky Skidmore | Email: [bskidmore@rogers.com](mailto:bskidmore@rogers.com) | Date completed: 3 Jun 2020 |
| --- | --- | --- | --- |
|  |  |  |  |

Do you wish to be acknowledged? (If yes, the review team will be advised to add an acknowledgement to any publications related to this work). Yes

The suggested acknowledgement is “We thank Becky Skidmore, MLS, Ottawa, ON for peer review of the MEDLINE search strategy.” [please edit to indicate your name, postnomials and institutional affiliation as you would like them presented].

|  | **1. TRANSLATION** |  | | |  |
| --- | --- | --- | --- | --- | --- |
| A -­‐No revisions |  |  |  |  |  |
| B -­‐ Revision(s) suggested |  |  |  |  |  |
| C -­‐ Revision(s) required | X |  |  |  |  |

If “B” or “C,” please provide an explanation or example:

I feel there is too much vocabulary missing to cover the required concepts adequately. For readability, would suggest concepts broken down into separate sets and joined together so AND/OR logic clearly stated.

**2. BOOLEAN AND PROXIMITY OPERATORS**

| A -­‐No revisions | X |
| --- | --- |
| B -­‐ Revision(s) suggested |  |
| C -­‐ Revision(s) required |  |

If “B” or “C,” please provide an explanation or example:

**3. SUBJECT HEADINGS**

| A -­‐No revisions |  |
| --- | --- |
| B -­‐ Revision(s) suggested |  |
| C -­‐ Revision(s) required | X |

If “B” or “C,” please provide an explanation or example:

Need MeSH:

Coronavirus Infections [mesh:noexp]; don’t explode but select and use subtree separately: Severe Acute Respiratory Syndrome [mesh]

Influenzavirus A[mesh]

Influenzavirus B[mesh]

See also suggestions under text-word searching.

**EML** – added mesh terms

**4. TEXT WORD SEARCHING**

| A -­‐No revisions |  |
| --- | --- |
| B -­‐ Revision(s)suggested |  |
| C -­‐ Revision(s) required | X |

If “B” or “C,” please provide an explanation or example:

For Elderberry concept, consider also black elders, sauco, elderflower*, Sambuci flos, "s nigra". Are there any brand names for existing supplements that can be included?

**EML** – I added elderflower*, “sambuci flos” and “s nigra”. I did not identify brand names that didn’t also use the term elderberry.

There is extensive vocabulary available for all population concepts. Have inserted examples of terminology (different interfaces depending on availability) for influenza, Covid-19, SARS, MERS from previously completed and PRESSed reviews, e.g.,

Covid-19 (can also search for PubMed or Ovid filters on Web e.g., <https://www.cebm.net/covid-19/what-is-the-evidence-for-use-of-macrolide-antobiotics-for-treatmetnof-covid-19/> - under Search Terms)

1 Coronavirus/ (2359)

2 Coronavirus Infections/ (8037)

3 (COVID-19 or COVID19).mp. (14882)

4 ((coronavirus* or corona virus*) and (hubei or wuhan or beijing or shanghai)).mp. (1422)

5 Wuhan virus*.mp. (6)

6 2019-nCoV.mp. (735)

7 (nCoV or n-CoV).mp. (774)

8 HCoV-19.mp. (7)

9 (SARS-CoV-2 or SARS-CoV2 or SARSCoV-2 or SARSCoV2).mp. (4582)

10 (novel coronavirus* or novel corona virus*).mp. (2481)

11 ((coronavirus* or corona virus*) adj2 "2019").mp. (3535)

12 ((coronavirus* or corona virus*) adj2 "19").mp. (739)

13 (coronavirus 2 or corona virus 2).mp. (4210)

14 (coronavirus* or corona virus*).ti. (9760)

**EML** – added SARS-COV-2 and 2019-nCoV, n-CoV; already had coronavirus OR coronaviruses

SARS:

Severe Acute Respiratory Syndrome[mesh] is a subtree of Respiratory Tract Infections[mesh] but still need text words:

Severe acute respiratory syndrome*[tiab]

**EML** – I used the text word ‘respiratory’ which will pull this in.

MERS:

Need text words for MERS-CoV, Middle East respiratory syndrome

**EML**- I used the text word ‘respiratory’ which will pull this in.

Influenza:

1 Influenza, Human/ (47631)

2 (influenza* or flu or grippe).tw,kf. (121344)

3 exp Influenzavirus A/ (42732)

4 exp Influenzavirus B/ (4150)

5 (H1N1 or PH1N1 or H3N2 or AH1N1 or "A(H1N1)" or "A/H1N1" or AH3N2 or "A(H3N2)" or "A/H3N2" or H5N1).tw,kf. (24959)

6 or/1-5 [INFLUENZA]

**EML** – added terms from line 5 above and influenza virus A and B

Common Cold:

Consider catarrh, coryza, possibly natural cold/colds

**EML** – added ‘colds’

Respiratory tract Infections:

Currently the MeSH is exploded as that is the default in PubMed. If you want all subtrees, that is fine but suggest including text words for some of the more important respiratory tract infections in this tree, e.g., bronchitis/bronchiolitis, pharyngitis, pneumonia, sinusitis if they are relevant.

**EML** – this review only pertains to influenza, common cold, SARS, MERS and COVID-19

Consider acronyms for LRTI, LRTIs, RTI, RTIs, i.e., (lower) respiratory tract infection(s)

**EML** – acronyms did not add any new references to results

Are you also interested in Respiratory Syncytial Viruses?

Otherwise consider NOT exploding Respiratory Tract Infections and only focussing on the infections of interest.

Respiratory Tract Infections[mesh:noexp]

Shock (Septic):

Consider rather than just “shock” as text word, be more specific, e.g., “endotoxic shock”, “toxic shock”. In working on various septic shock reviews developing guidelines, the following terminology was considered relevant to describing septic shock patients (*Cochrane Library syntax*):

**EML** – added sepsis, bacteremia, fengemia, parasitemia, viremia

#1 [mh ^Sepsis] 1647

#2 [mh Bacteremia] 851

#3 [mh Fungemia] 75

#4 [mh "Shock, Septic"] 538

#5 [mh Parasitemia] 306

#6 [mh Viremia] 314

#7 (sepsis or septic*):ti,ab,kw 8700

#8 ((toxic or endotoxic) next shock*):ti,ab,kw 31

#9 (blood near/3 (infection* or poisoning)):ti,ab,kw 1015

#10 (bloodstream* near/3 (infection* or poisoning)):ti,ab,kw 627

#11 (pyaemia* or pyemia* or pyohemia*):ti,ab,kw 5

#12 (bacteremia* or bacteraemia*):ti,ab,kw 1920

#13 (fungemia* or fungaemia* or candidemia* or candidaemia*):ti,ab,kw 224

#14 (parasitemia* or parasitaemia*):ti,ab,kw 1008

#15 (viremia* or viraemia*):ti,ab,kw 990

#16 [mh ^"Bacterial Infections"] 3072

#17 (bacterial next infection*):ti,ab,kw 5135

#18 [mh "Fasciitis, Necrotizing"] 4

#19 ((necrotizing or necrotising) next fasciitis):ti,ab,kw 37

#20 ("flesh-eating" next (disease* or bacteria*)):ti,ab,kw 0

#21 [mh "Intestinal Perforation"] 151

#22 (perforat* near/5 (bowel* or intestin* or anus* or anal or anally or appendi* or cecum* or colon or colonic or colons or duoden* or ileum* or jejunum* or rectum* or rectal or rectally)):ti,ab,kw 852

#23 [mh Peritonitis] 532

#24 (peritonitis or (peritone* near/5 (infection* or inflammation*))):ti,ab,kw 1569

#25 [mh Meningitis] 546

#26 (meningiti* or meningoencephaliti* or (meningo next encephaliti*)):ti,ab,kw 1551

#27 ([mh "Critical Illness"] or [mh "Critical Care"] or [mh "Emergency Service, Hospital"] or [mh "Intensive Care Units"]) and ([mh Infection] or infection*:ti,ab,kw) 1571

#28 (infection* near/5 (acute* or critical* or emergency or emergencies or "intensive care" or "life-threatening" or serious* or severe*)):ti,ab,kw 6808

#29 (Ulbricht et al. -#28) 25638

Cytokines:

Consider also interleukin. Are any of these subtrees available? If so, need text words.

**EML** - Review looks at cytokines including including interferons (IFNs), interleukins (ILs), chemokines, colony-stimulating factors (CSFs), and tumor necrosis factor (TNF-alpha) – terms added


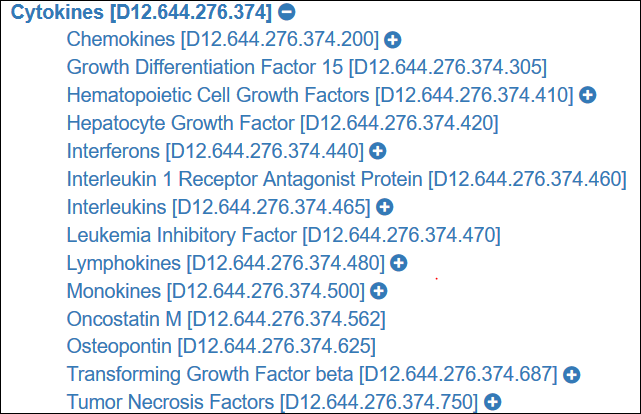


**5. SPELLING, SYNTAX, AND LINE NUMBERS**

| A -­‐No revisions | X |
| --- | --- |
| B -­‐ Revision(s)suggested |  |
| C -­‐ Revision(s) required |  |

If “B” or “C,” please provide an explanation or example:

**6. LIMITS AND FILTERS**

| A -­‐No revisions |  |
| --- | --- |
| B -­‐ Revision(s) suggested | X |
| C -­‐ Revision(s) required |  |

If “B” or “C,” please provide an explanation or example:

Consider removing Animal-only studies

OVERALL EVALUATION (Note: If one or more “revision required” is noted above, the response below must be “revisions required”.)

| A -­‐No revisions |  |
| --- | --- |
| B -­‐ Revision(s) suggested |  |
| C -­‐ Revision(s) required | X |

Additional comments:

Noted errors are mostly errors of omission. Systematic review searches require sensitive searching, with extensive vocabulary to represent all the concepts.

Ulbricht, C., E. Basch, L. Cheung, H. Goldberg, P. Hammerness, R. Isaac, K. P. Khalsa, A. Romm, I. Rychlik, M. Varghese, W. Weissner, R. C. Windsor, and J. Wortley. 2014. "An evidence-based systematic review of elderberry and elderflower (Sambucus nigra) by the Natural Standard Research Collaboration." *J Diet Suppl* 11 (1):80-120. doi: 10.3109/19390211.2013.859852.
