# Supplementary material for: Elderberry for prevention and treatment of viral respiratory illnesses: a systematic review
Source: BMC Complement Med Ther. 2021 Apr 7;21:112. doi: 10.1186/s12906-021-03283-5 (PMC8026097; doi:10.1186/s12906-021-03283-5)
Supplement: Supplementary file 2 — Additional file 2. [file 12906_2021_3283_MOESM2_ESM.docx]

References to excluded studies:

Anonymous 1998

Anonymous. Anecdotal reports: elderberry extract plus chondroitin and glucosamine sulfate and Thy-mate reduces viral load to non-detectable levels in 10 days. Positive health news 1998;No 17:7-11.

Bagchi 2004

Bagchi D, Sen C K, Bagchi M, Atalay M. Anti-angiogenic, antioxidant, and anti-carcinogenic properties of a novel anthocyanin-rich berry extract formula. Biochemistry (Mosc) 2004;69(1):75-80, 1 p preceding 75.

Bagchi 2004a

Bagchi D, Sen C K, Bagchi M, Atalay M. Review: Antiangiogenic, antioxidant, and anticarcinogenic properties of a novel anthocyanin-rich berry extract formula. Biokhimiya 2004;69(1):95-102.

Balkan 2017

Balkan İ A, İlter Akülke A Z, Bağatur Y, Telci D, Gören A C, Kırmızıbekmez H, et al. Sambulin A and B, non-glycosidic iridoids from Sambucus ebulus, exert significant in vitro anti-inflammatory activity in LPS-induced RAW 264.7 macrophages via inhibition of MAPKs's phosphorylation. Journal of Ethnopharmacology 2017;206:347-52.

Barak 2001

Barak V, Halperin T, Kalickman I. The effect of Sambucol, a black elderberry-based, natural product, on the production of human cytokines: I. Inflammatory cytokines. European Cytokine Network 2001;12(2):290-6.

Barak 2002

Barak V, Birkenfeld S, Halperin T, Kalickman I. The effect of herbal remedies on the production of human inflammatory and anti-inflammatory cytokines. Israel Medical Association Journal 2002;4(11 SUPPL.):919-22.

Bitsch 2004

Bitsch I, Janssen M, Netzel M, Strass G, Frank T. Bioavailability of anthocyanidin-3-glycosides following consumption of elderberry extract and blackcurrant juice. 2004;42(5):293‐300.

Carneiro 2019

Carneiro N V Q, Silva H B F D, Silva R R D, Carneiro T C B, Costa R S, Pires A O, et al. Sambucus australis Modulates Inflammatory Response via Inhibition of Nuclear Factor Kappa B (NF-kB) in vitro. Anais da Academia Brasileira de Ciencias 2019;91(1):e20170831.

Chen 2002

Chen Y, Peumans W J, Van Damme E J M. The Sambucus nigra type-2 ribosome-inactivating protein SNA-I' exhibits in planta antiviral activity in transgenic tobacco. 2002;516(1‐3):27‐30.

Chen 2014

Chen C, Zuckerman D M, Brantley S, Sharpe M, Childress K, Hoiczyk E, et al. Sambucus nigra extracts inhibit infectious bronchitis virus at an early point during replication. BMC Veterinary Research 2014;10(24):(16 January 2014).

Cordier 2003

Cordier H. Common cold in children: A case for "black elder" [1]. MMW-Fortschritte der Medizin 2003;145(15):12.

Das 2011

Das S. Exploitation of antiviral activity of traditionally used medicinal plants. Journal of Antivirals and Antiretrovirals 2011;2011((Das S.) Noida Institute of Engineering and Technology, India).

David 2014

David L, Moldovan B, Vulcu A, Olenic L, Perde-Schrepler M, Fischer-Fodor E, et al. Green synthesis, characterization and anti-inflammatory activity of silver nanoparticles using European black elderberry fruits extract. Colloids Surf B Biointerfaces 2014;122:767-77.

de Ferrars 2014

de Ferrars R M, Cassidy A, Curtis P, Kay C D. Phenolic metabolites of anthocyanins following a dietary intervention study in post-menopausal women. 2014;58(3):490‐502.

Della 2019

Della Volpe A, Ricci G, Ralli M, Gambacorta V, De Lucia A, Minni A, et al. The effects of oral supplements with Sambucus nigra, Zinc, Tyndallized Lactobacillus acidophilus (H122), Arabinogalactans, vitamin D, vitamin E and vitamin C in otitis media with effusion in children: a randomized controlled trial. 2019;23(14):6360‐6370.

Denzler 2010

Denzler KL, Waters R, Jacobs BL, Rochon Y, Langland JO. Regulation of inflammatory gene expression in PBMCs by immunostimulatory botanicals. PLoS One 2010;5(9):e12561.

Draganova 2013

Draganova V. Preventive and therapeutic use of natural molecular complexes with immunoregulatory effect during childhood. Pediatriya 2013;53(4):73-5.

Elrod 2015

Elrod S, Greenspan P. Effect of Muscadine Juice and Elderberry Preparations on Wild-Type H1N1 Influenza Virus-Induced Cytotoxicity. Faseb Journal 2015;29:1-1.

EUCTR2009-016682-28-DE [Other identifier: ACTRN12615000506594]

EUCTR2009-016682-28-DE. A multi-centre, double-blind, placebo-controlled, randomised, parallel group study to assess the efficacy and safety of a herbal medicinal product (dry extract BNO-1016) in patients with acute rhinosinusitis. https://www.clinicaltrialsregister.eu/ctr-search/trial/2008-002794-13/DE (submitted on 2009/11/04).

EUCTR2015-001952-31-DE [Other identifier: ACTRN12615000506594]

EUCTR2015-001952-31-DE. A multicenter, randomized, double-blind, placebo-controlled, parallel-group clinical trial to assess efficacy and safety of the herbal medicinal product Sinupret extract coated tablets in patients with chronic rhinosinusitis. https://www.clinicaltrialsregister.eu/ctr-search/trial/2015-001952-31/DE (submitted on 2015/11/27).

Frank 2007

Frank T, Janssen M, Netzet G, Christian B, Bitsch I, Netzel M. Absorption and excretion of elderberry (Sambucus nigra L.) anthocyanins in healthy humans. 2007;29(8):525‐533.

Frechet 2015

Frechet M, Valenti L, Markioli P, Lafitte P, Maccario F, Nicolay J. Inhibition of adhesion proteins expression in human dermal microvascular endothelial cells exposed to TNF-alpha by a Sambucus nigra extract. Journal of Investigative Dermatology 2015;135:S94.

Frøkiær 2012

Frøkiær H, Henningsen L, Metzdorff S B, Weiss G, Roller M, Flanagan J, et al. Astragalus Root and Elderberry Fruit Extracts Enhance the IFN-beta Stimulatory Effects of Lactobacillus acidophilus in Murine-Derived Dendritic Cells. Plos One 2012;7(10):10.

Gessner 2010

Gessner A. Sinupret®-an updated pharmacological view on a success story. Planta Medica 2010;76(12).

Harokopakis 2006

Harokopakis E, Albzreh MH, Haase EM, Scannapieco FA, Hajishengallis G. Inhibition of proinflammatory activities of major periodontal pathogens by aqueous extracts from elder flower (Sambucus nigra). J Periodontol 2006;77(2):271-9.

Hasaninejad-Farahani 2015

Hasaninejad-Farahani A, Shahsavandi S, Ebrahimi M. The potential inhibitory effect of Sambucus nigra fruit on early replication of influenza virus. Journal of Isfahan Medical School 2015;33(352):1607-17.

He 2004

He L. A review of research of secretolytics, Sinupret drop. Chinese Journal of New Drugs 2004;13(Mar):272-4.

Ivanova 2013

Ivanova D, Tasinov O, Nazifova N, Kiselova-Kaneva Y. Sambucus ebulus fruit extracts reduce TNFá and il6 expression elevated in ethanol-treated 3t3-l1 preadipocytes. Annals of Nutrition and Metabolism 2013;63):294.

Jabbari 2017

Jabbari M, Daneshfard B, Emtiazy M, Khiveh A, Hashempur M H. Biological Effects and Clinical Applications of Dwarf Elder (Sambucus ebulus L): A Review. Journal of Evidence-Based Complementary and Alternative Medicine 2017;22(4):996-1001.

Jareoncharsri 2002

Jareoncharsri P, Bunnag C, Tunsuriyawong P, Atipas M, Voraprayoon S. An open-label study of the efficacy and safety of a herbal secretolytic preparation as a sole therapy in patients with sinusitis. Siriraj Hospital Gazette 2002;54(7):379-86.

Jund 2013

* Jund R, Mondigler M, Steindl H, Stammer H, Stierna P, Bachert C. Accelerated remission of symptoms of acute viral rhinosinusitis with a dry extract of five herbal drugs (BNO 1016). 2013;79(13).

Jund R, Mondigler M, Steindl H, Stammer H, Stierna P, Bachert C. Clinical efficacy of a dry extract of five herbal drugs in acute viral rhinosinusitis. 2012;50(4):417‐426.

Knudsen 2015

Knudsen B F, Kaack K V. A review of human health and disease claims for elderberry (Sambucus nigra) fruit. Acta Horticulturae 2015;(No.1061):121-31.

Kreft 2012

Kreft S. Selected herbal medicinal products in otolaryngology. Farmacevtski Vestnik 2012;63(2):109-11.

Kronbichler 2020

Kronbichler A, Effenberger M, Eisenhut M, Lee K H, Shin J I. Seven recommendations to rescue the patients and reduce the mortality from COVID-19 infection: An immunological point of view. Autoimmun Rev 2020;19(7):102570.

Lasku 2010

Lasku A, Denzler K, Jacobs B, Rochon Y, Langland J, Waters R, et al. Genetic expression analysis of immunostimulatory botanicals on human peripheral blood mononuclear cells associated with inflammatory processes. FASEB Journal 2010;24.

Li 2017

Li M, Shengliang Y, Xianda Z, Minxia H, Dong L, Changqing L. Inhibition of inflammatory cytokines by IVIG is dependent of IGG-FC sialylation in human monocytic THP-1 cells. Vox Sanguinis 2017;112:88.

Lin 2019

Lin P, Hwang E, Ngo HTT, Seo SA, Yi TH. Sambucus nigra L. ameliorates UVB-induced photoaging and inflammatory response in human skin keratinocytes. Cytotechnology 2019;71(5):1003-17.

Luo 2020

Luo Hui, Tang Qiao-Ling, Shang Ya-Xi, Liang Shi-Bing, Yang Ming, Robinson Nicola, et al. Can Chinese Medicine Be Used for Prevention of Corona Virus Disease 2019 (COVID-19)? A Review of Historical Classics, Research Evidence and Current Prevention Programs. Chin J Integr Med 2020.

Maleknia 2016

Maleknia S D, Downard K M. New anthocyanins from black elderberry of inhibitory potential revealed by mass spectrometry. Natural Products Journal 2016;6(2):94-102.

Matrosovich 2003

Matrosovich M, Matrosovich T, Carr J, Roberts N A, Klenk H D. Overexpression of the α-2,6-sialyltransferase in MDCK cells increases influenza virus sensitivity to neuraminidase inhibitors. Journal of Virology 2003;77(15):8418-25.

Maurel 2016

Maurel S, Libon C, Pourtau S, Issac C, Haddioui L, Ripoll C. Immunomodulatory in vitro and in vivo effects of lactobacillus rhamnosus and elderberry extract alone and in combination. Journal of Clinical Gastroenterology 2016;50(Supplement 2):S199.

Neves 2019

Neves D, Valentão P, Bernardo J, Oliveira M C, Ferreira J M G, Pereira D M, et al. A new insight on elderberry anthocyanins bioactivity: modulation of mitochondrial redox chain functionality and cell redox state. Journal of Functional Foods 2019;56:145-55.

Nilsson 2017

Nilsson A, Salo I, Plaza M, Björck I. Effects of a mixed berry beverage on cognitive functions and cardiometabolic risk markers; A randomized cross-over study in healthy older adults. PLoS One 2017;12(11):e0188173.

Nunes 2011

Nunes T, Rocha J F, Vaz-da-Silva M, Falcão A, Almeida L, Soares-da-Silva P. Pharmacokinetics and tolerability of etamicastat following single and repeated administration in elderly versus young healthy male subjects: an open-label, single-center, parallel-group study. 2011;33(6):776‐791.

Olejnik 2015

Olejnik A, Kowalska K, Olkowicz M, Rychlik J, Juzwa W, Myszka K, et al. Anti-inflammatory effects of gastrointestinal digested Sambucus nigra L. fruit extract analysed in co-cultured intestinal epithelial cells and lipopolysaccharide-stimulated macrophages. Journal of Functional Foods 2015;19(Part A):649-60.

Ouyang 2020

Ouyang Yabo, Yin Jiming, Wang Wenjing, Shi Hongbo, Shi Ying, Xu Bin, et al. Down-regulated gene expression spectrum and immune responses changed during the disease progression in COVID-19 patients. Clin Infect Dis 2020.

Putra 2019

Putra W E, Salma W O, Rifa'i M. Anti-inflammatory activity of sambucus plant bioactive compounds against TNF-α and trail as solution to overcome inflammation associated diseases: The insight from bioinformatics study. Natural Product Sciences 2019;25(3):215-21.

Ren 2020

Ren Yue, Yao Mei-Cun, Huo Xiao-Qian, Gu Yu, Zhu Wei-Xing, Qiao Yan-Jiang, et al. [Study on treatment of "cytokine storm" by anti-2019-nCoV prescriptions based on arachidonic acid metabolic pathway]. Zhongguo Zhong Yao Za Zhi 2020.

Schwaiger 2011

Schwaiger S, Zeller I, Pölzelbauer P, Frotschnig S, Laufer G, Messner B, et al. Identification and pharmacological characterization of the anti-inflammatory principal of the leaves of dwarf elder (Sambucus ebulus L.). Journal of Ethnopharmacology 2011;133(2):704-9.

Serkedjieva 1990

Serkedjieva J, Manolova N, Zgorniak-Nowosielska I, Zawilinska B, Grzybek J. Antiviral activity of the infusion (SHS-174) from flowers of Sambucus nigra L., aerial parts of Hypericum perforatum L., and roots of Saponaria officinalis L. against influenza and herpes simplex viruses. Phytotherapy Research 1990;4(3):97-100.

Sun 2013

Sun G Y, Chuang D Y, Zong Y, Jiang J, Simonyi A, Gu Z, et al. Botanical polyphenols as potential therapeutic agents for mitigating oxidative/ nitrosative and inflammatory responses in microglial cells. Folia Neuropathologica 2013;51(4):378.

Tasinov 2014

Tasinov O, Kiselova-Kaneva Y, Ivanova D. Sambucus ebulus L. tea consumption affects leptin and adiponectin levels in healthy volunteers. Obesity Facts 2014;7:129.

Tesche 2008

Tesche S, Metternich F, Sonnemann U, Engelke J C, Dethlefsen U. The value of herbal medicines in the treatment of acute non-purulent rhinosinusitis. Results of a double-blind, randomised, controlled trial. 2008;265(11):1355‐1359.

Timoshenko 1995

Timoshenko A V, Cherenkevich S N. [H2O2 generation and human neutrophil aggregation as affected by lectins]. Gematol Transfuziol 1995;40(4):32-5.

Torabian 2019

Torabian G, Valtchev P, Adil Q, Dehghani F. Anti-influenza activity of elderberry (Sambucus nigra). Journal of functional foods 2019;54:353-60.

Vimalanathan 2013

Vimalanathan S, Schoop R, Pleschka S, Hudson J. Synergistic inhibition of Influenza replication cycle with Echinacea purpurea and Sambucus nigra. Planta Medica 2013;79(13).

Vishnyakov 2013

Vishnyakov V V, Sinkov D E. Herbal medicine as add-on therapy in acute Rhinosinusitis: results of an open randomized cohort study with the herbal combination Sinupret®. 2013;34(6):262‐265.

Vogl 2013

Vogl S, Picker P, Mihaly-Bison J, Fakhrudin N, Atanasov AG, Heiss EH, et al. Ethnopharmacological in vitro studies on Austria's folk medicine--an unexplored lore in vitro anti-inflammatory activities of 71 Austrian traditional herbal drugs. J Ethnopharmacol 2013;149(3):750-71.

Walker 2013

Walker JM, Maitra A, Walker J, Ehrnhoefer-Ressler MM, Inui T, Somoza V. Identification of Magnolia officinalis L. bark extract as the most potent anti-inflammatory of four plant extracts. American Journal of Chinese Medicine 2013;41(3):531-544.

Wang 2019

Wang Z Z, Zeng M, Wang H, Song J, Liu Z. Evidence for a role of ST6GAL1 in goblet cell hyperplasia in allergic rhinitis patients. Journal of Allergy and Clinical Immunology 2019;143(2):AB288.

Werz 2013

Werz O, Seifert S, Wosikowski K, Haunschild J. Anti-viral and anti-inflammatory efficacies of Sinupret® dry extract BNO 1011 rationalise its therapeutic use in acute rhinosinusitis. Planta Medica 2013;79(13).

Williams 2017 [Other identifier: ACTRN12614000079640]

Williams EJ, Baines KJ, Berthon BS, Wood LG. Effects of an encapsulated fruit and vegetable juice concentrate on obesity-induced systemic inflammation: a randomised controlled trial. Nutrients 2017;9(2):116.

Wiesenauer 2004

Wiesenauer M, Keller G. Homeopathy - Did you know? Transferred nasal breathing. Treatment of common cold in infants with Sambucus nigra D3. Deutsche Apotheker Zeitung 2004;144(23):61.

Yang 2015

Yang B, Lin X, Yang C, Tan J, Li W, Kuang H. Sambucus Williamsii Hance Promotes MC3T3-E1 Cells Proliferation and Differentiation via BMP-2/Smad/p38/JNK/Runx2 Signaling Pathway. Phytotherapy Research 2015;29(11):1692-9.

Yarnell 2018

Yarnell E. Herbs for Viral Respiratory Infections. Alternative and Complementary Therapies 2018;24(1):35-43.

Yeşilada 1997

Yeşilada E, Üstün O, Sezik E, Takaishi Y, Ono Y, Honda G. Inhibitory effects of Turkish folk remedies on inflammatory cytokines: Interleukin-1α, interleukin-1β and tumor necrosis factor α. Journal of Ethnopharmacology 1997;58(1):59-73.

Youdim 2000

Youdim K A, Martin A, Joseph J A. Incorporation of the elderberry anthocyanins by endothelial cells increases protection against oxidative stress. Free Radical Biology and Medicine 2000;29(1):51-60.

Zhou 2014

Zhou S S. Antiviral activity of AV-001 against influenza and three common cold viruses. Journal of Natural Remedies 2014;14(2):174-8.

Zielińska-Wasielica 2019

Zielińska-Wasielica J, Olejnik A, Kowalska K, Olkowicz M, Dembczyński R. Elderberry (Sambucus nigra L.) fruit extract alleviates oxidative stress, insulin resistance, and inflammation in hypertrophied 3T3-L1 adipocytes and activated RAW 264.7 macrophages. Foods 2019;8(8):326.

Reasons for exclusion:

Anonymous 1998 Reason for exclusion: Unable to obtain

Bagchi 2004 Reason for exclusion: Wrong outcomes

Bagchi 2004a Reason for exclusion: Wrong study design

Balkan 2017 Reason for exclusion: In vitro study

Barak 2001 Reason for exclusion: In vitro study

Barak 2002 Reason for exclusion: In vitro study

Bitsch 2004 Reason for exclusion: Wrong outcomes

Carneiro 2019 Reason for exclusion: In vitro study

Chen 2002 Reason for exclusion: In vitro study

Chen 2014 Reason for exclusion: Wrong indication

Cordier 2003 Reason for exclusion: Wrong study design

Das 2011 Reason for exclusion: Abstract with no outcomes

David 2014 Reason for exclusion: In vitro study

de Ferrars 2014 Reason for exclusion: Wrong outcomes

Della 2019 Reason for exclusion: Wrong patients

Denzler 2010 Reason for exclusion: In vitro study

Draganova 2013 Reason for exclusion: Unable to obtain

Elrod 2015 Reason for exclusion: In vitro study

EUCTR2009-016682-28-DE Reason for exclusion: Wrong patient population

EUCTR2015-001952-31-DE Reason for exclusion: Wrong patient population

Frank 2007 Reason for exclusion: Wrong outcomes

Frechet 2015 Reason for exclusion: Abstract with no outcomes

Frøkiær 2012 Reason for exclusion: In vitro study

Gessner 2010 Reason for exclusion: Wrong study design

Harokopakis 2006 Reason for exclusion: In vitro study

Hasaninejad-Farahani 2015 Reason for exclusion: Wrong outcomes

He 2004 Reason for exclusion: Wrong study design

Ivanova 2013 Reason for exclusion: In vitro study

Jabbari 2017 Reason for exclusion: Wrong study design

Jareoncharsri 2002 Reason for exclusion: Unable to obtain

Jund 2013 Reason for exclusion: Wrong patients

Knudsen 2015 Reason for exclusion: Wrong study design

Kreft 2012 Reason for exclusion: Wrong outcomes

Kronbichler 2020 Reason for exclusion: Wrong study design

Lasku 2010 Reason for exclusion: Abstract with no outcomes

Li 2017 Reason for exclusion: Abstract with no outcomes

Lin 2019 Reason for exclusion: In vitro study

Luo 2020 Reason for exclusion: Wrong study design

Maleknia 2016 Reason for exclusion: Wrong outcomes

Matrosovich 2003 Reason for exclusion: Wrong intervention

Maurel 2016 Reason for exclusion: In vitro study

Neves 2019 Reason for exclusion: Wrong outcomes

Nilsson 2017 Reason for exclusion: Wrong intervention

Nunes 2011 Reason for exclusion: Wrong intervention

Olejnik 2015 Reason for exclusion: In vitro study

Ouyang 2020 Reason for exclusion: Wrong intervention

Putra 2019 Reason for exclusion: Wrong setting

Ren 2020 Reason for exclusion: Wrong intervention

Schwaiger 2011 Reason for exclusion: Wrong outcomes

Serkedjieva 1990 Reason for exclusion: Wrong outcomes

Sun 2013 Reason for exclusion: Wrong intervention

Tasinov 2014 Reason for exclusion: Abstract with no outcomes

Tesche 2008 Reason for exclusion: Wrong patient population

Timoshenko 1995 Reason for exclusion: Unable to obtain

Torabian 2019 Reason for exclusion: In vitro study

Vimalanathan 2013 Reason for exclusion: Wrong outcomes

Vishnyakov 2013 Reason for exclusion: Wrong patient population

Vogl 2013 Reason for exclusion: In vitro study

Walker 2013 Reason for exclusion: In vitro study

Wang 2019 Reason for exclusion: Abstract with no outcomes

Werz 2013 Reason for exclusion: Abstract with no outcomes

Williams 2017 Reason for exclusion: Wrong intervention

Wlesenauer 2004 Reason for exclusion: Unable to obtain

Yang 2015 Reason for exclusion: In vitro study

Yarnell 2018 Reason for exclusion: Wrong study design

Yeşilada 1997 Reason for exclusion: In vitro study

Youdim 2000 Reason for exclusion: In vitro study

Zhou 2014 Reason for exclusion: Wrong outcomes

Zielińska-Wasielica 2019 Reason for exclusion: In vitro study
