# Supplementary material for: Elderberry for prevention and treatment of viral respiratory illnesses: a systematic review
Source: BMC Complement Med Ther. 2021 Apr 7;21:112. doi: 10.1186/s12906-021-03283-5 (PMC8026097; doi:10.1186/s12906-021-03283-5)
Supplement: Supplementary file 3 — Additional file 3. [file 12906_2021_3283_MOESM3_ESM.docx]

| **Kong 2009** |
| --- |
| **Study details**  SPONSORSHIP SOURCE  sponsored by HerbalScience Singapore Pte. Ltd  "Trial conducted by Medical Personnel at Shanghai Construction Technical College, China, on behalf of Herbal Science Singapore Pte. Ltd., Singapore."  COUNTRY  China  SETTING  Outpatient  COMMENTS  Treatment study for persons with influenza symptoms.  Year(s) conducted: 2009  **Author's contact details**  NAME  Fan‐kun Kong, PhD.  INSTITUTION  Shanghai Construction Technical College  EMAIL  None reported  ADDRESS  China  **COIs**  “[T]he author admits no competing interests.” |
| **Methods**  DESIGN  Randomized controlled trial, double-blind  GROUP  Parallel group  HOW WERE ADVERSE EVENTS ASSESSED?  No mention of specific ascertainment however lack of side effects with elderberry was mentioned: "In the clinical study here, the proprietary elderberry extract was shown to be safe as no patients receiving the proprietary elderberry extract reported any adverse events including nausea and vomiting, which are two adverse‐events common in anti‐viral treatments." |
| **Population**  INCLUSION CRITERIA  "Volunteers (age ranged 16 to 60 years) presenting flu symptoms for less than 24 hours, and otherwise healthy individuals were included in the clinical study. The admitted participants had at least three of the following symptoms: fever, headache, muscle aches, coughing, mucus discharge and nasal congestion."  EXCLUSION CRITERIA  "Patients who suffered from chronic diseases, were suspected of having a bacterial infection, participated in another clinical trial, or recently received flu medication, antiviral therapy, or influenza vaccination were excluded from the study. Pregnant and breastfeeding women were also excluded from the study."  GROUP DIFFERENCES  "There were no obvious differences in demographic characteristics between the groups (p>0.05) (Table 1). At their first visit, the majority of patients from both groups complained of headache, muscle aches, and nasal congestion, while fewer patients had fever, cough and nasal mucus discharge, all indicative symptoms of an influenza infection (Table 2)." |

| **Kong 2009** | | | |
| --- | --- | --- | --- |
| **Baseline Characteristics** |  |  |  |
| **Characteristic** | **Elderbery** | **Placebo** | **Overall** |
| Sample size | 32 | 32 | 64 |
| Age in years | Range 20‐55 years; Mean 40 years | Range 27‐59 years; Mean 40.1 years | Range 20-59 years; Mean 40 years |
| Male/Female numbers | 17//15 | 17/15 | 34/30 |
| Race/Ethnicity (if reported) (n) | NR | NR | NR |
| Socioeconomic status (if reported) | NR | NR | NR |
| Comorbidities and known risk factors: number smokers | NR | NR | NR |
| Influenza A (n) | NR | NR | NR |
| Influenza B (n) | NR | NR | NR |
| Duration of illness at study entry (first treatment) (mean with units) | <24 hours | <24 hours | <24 hours |
| Fever at study entry (n) | 15 | 9 | 24 |
| Cough (any) at study entry (n) | 16 | 16 | 32 |
| Dry cough at study entry (n) | NR | NR | NR |
| Productive cough at study entry (n) | NR | NR | NR |
| Headache at study entry (n) | 32 | 32 | 64 |
| Nasal congestion at study entry (n) | 32 | 28 | 60 |
| Muscle aches at study entry (n) | 31 | 30 | 61 |
| Mucus discharge at study entry (n) | 16 | 11 | 27 |
| Fatigue at study entry (n) | NR | NR | NR |
| Sore throat at study entry (n) | NR | NR | NR |

| **Kong 2009** |  |  |
| --- | --- | --- |
| **Intervention(s) and Comparator(s)** |  |  |
| **Characteristic** | **Elderbery** | **Placebo** |
| Name of commercial product | Not described; unclear whether it is/was commercially available | N/A |
| Type of product | Lozenge | Lozenge |
| Formulation of product | "Each lozenge contained 175 mg of the proprietary elderberry extract plus non‐active ingredients (maltodextrin, dextrose, fructose, silica, citric acid, natural flavors, cyclodextrin and magnesium stearate). "  Unclear if berry or other plant part used. While *Sambucus nigra* L. mentioned in the Introduction, the study idid not directly specify this was used to create the proprietary extract. | "A placebo lozenge that was identical in appearance, taste, and composition except that there was no elderberry extract included, was supplied in similar packaging." |
| Standardization and manufacturing | Not described | Not described |
| Dose of product | 1 slow-dissolve lozenge with 175 mg elderberry extract | 1 slow-dissolve lozenge without extract |
| Frequency of dose | 4 times per day (before each meal and before bed) | 4 times per day (before each meal and before bed) |
| Duration of treatment | 2 days | 2 days |
| Concomitant medications | Not mentioned | |

| **Kong 2009** |  |  |
| --- | --- | --- |
| **Outcomes** |  |  |
| Percentage with baseline symptoms of headache, nasal congestion, muscle aches, coughing, mucus discharge or fever, and the number or percentage with improved symptoms at 24 and 48 hours. The mean self-assessed VAS scale (from 0 to 10 where 10 is worse symptom) measure for each symptom at baseline, one day and two days. | | |

| **Rauš 2015** |
| --- |
| **Identification**  **Study details**  SPONSORSHIP SOURCE  A. Vogel Bioforce AG, Roggwil, Switzerland  COUNTRY  Czech Republic  SETTING  Outpatient  COMMENTS  Year(s) conducted: 2011-2013  Treatment study for persons with Influenza symptoms.  **Author's contact details**  NAME  Roland Schoop  INSTITUTION  A. Vogel Bioforce AG  EMAIL  r.schoop@bioforce.ch  ADDRESS  A. Vogel Bioforce AG, Grünaustrasse, 9325 Roggwil (TG), Switzerland.  **COIs**  “R. Schoop is an employee of Bioforce AG, and K. Rauš and P. Klein have received honorarium funds from the study sponsor. The authors have indicated that they have no other conflicts of interest regarding the content of this article.” |
| **Methods**  DESIGN  Randomized controlled trial, double-blinded  GROUP  Parallel group  HOW WERE ADVERSE EVENTS ASSESSED?  "To detect adverse events (AEs) patients were asked, “Did you experience any unusual or unexpected symptom since your last visit, apart from the symptoms recorded in the diary?" Changes in concomitant diseases and medications as well as laboratory parameters were also taken into account before judgment of AE causality." |
| **Population**  INCLUSION CRITERIA  Participants were required to have a clinical diagnosis of influenza. "Clinical diagnosis of influenza was based on the presence of at least 1 respiratory symptom (eg, cough, sore throat, or nasal symptom), 1 constitutional symptom (eg, headache, malaise, myalgia, sweats and/or chills, and fatigue), and fever (>=37.81C), with symptoms not present for more than 48 hours. A negative pregnancy test, body weight >40 kg, good general health, and a signed informed consent were prerequisites for inclusion."  EXCLUSION CRITERIA  "The following exclusion criteria were applied: intake of anti-microbial agents during the past month; influenza vaccination during the past 12 months; suspected bacterial infection; bronchitis; intake of steroid or immune-suppressive medication; pregnancy or breastfeeding; chronic cardiac diseases; known endocrine disorders like diabetes mellitus; liver, kidney, and respiratory disorders; asthma; and serious chronic diseases influencing absorption, metabolism, and the elimination of the investigational product. Patients with known AIDS, autoimmune disease, as well as clinically significant chronic disease; illness requiring hospitalization; known allergy to plants of the Compositae family, paracetamol, or dextromethorphan; psychiatric disorders; neurologic and neurodevelopment conditions; planned surgical intervention during the trial period; alcohol or drug abuse; nicotine addiction; and participation in another trial were excluded. Women without effective contraception were also excluded."  GROUP DIFFERENCES  "Patients in the 2 treatment groups were comparable with regard to age, body weight, body height, body mass index, and sex distribution (Table 1)." "Physical baseline values, including heart rate and blood pressure, were similar in the treatment groups." |

| **Rauš 2015** | | | |
| --- | --- | --- | --- |
| **Baseline Characteristics** |  |  |  |
| **Characteristic** | **Echinacea/Elderberry** | **Oseltamivir** | **Overall** |
| Sample size* | 203 | 217 | 420 |
| Age in years | Mean (sd) 37.7 (13.9) | Mean (sd) 36.7 (13.1) | Mean (sd) 37.18 (13.49)* |
| Male/Female numbers | 109/94 | 101/116 | 210/210 |
| Race/Ethnicity (if reported) | “all Caucasian” | “all Caucasian” | “all Caucasian” |
| Socioeconomic status (if reported) | NR | NR | NR |
| Comorbidities and known risk factors: number smokers | NR | NR | NR |
| Influenza A (n)* | NR | NR | 41/420 had positive tests for influenza A and/or Influenza B; n=10 with influenza type A [H3], n=17 with nontypeable influenza A, n=1 with coinfection of influenza A and B** |
| Influenza B (n)* | NR | NR | 41/420 had positive tests for influenza A and/or Influenza B; n=13 with influenza type B, n=1 with coinfection of influenza A and B** |
| Duration of illness at study entry (first treatment) (mean with units) | NR | NR | Inclusion criterion was symptoms present ≤48 hrs |
| Fever at study entry (n) | 69.27% moderate/severe symptom | 69.03% moderate/severe symptom | NR |
| Cough (any) at study entry (n) | 82.82% moderate/severe symptom | 76.15% moderate/severe symptom | NR |
| Dry cough at study entry (n) | NR | NR | NR |
| Productive cough at study entry (n) | NR | NR | NR |
| Headache at study entry (n) | NR | NR | NR |
| Nasal congestion at study entry (n) | NR | NR | NR |
| Muscle aches at study entry (n) | NR | NR | NR |
| Mucus discharge at study entry (n) | NR | NR | NR |
| Fatigue at study entry (n) | NR | NR | NR |
| Sore throat at study entry (n) | NR | NR | NR |

*Calculated from information reported in study.

**” “The rates of recovery of patients with confirmed influenza virus infection were very similar to those in clinically diagnosed influenza patients”

| **Rauš 2015** |  |  |
| --- | --- | --- |
| **Intervention(s) and Comparator(s)** |  |  |
| **Characteristic** | **Echinacea/Elderbery** | **Oseltamivir** |
| Name of commercial product | Echinaforce Hotdrink (+ oseltamivir placebo) | Tamiflu (+ Echinaforce Hotdrink placebo) |
| Type of product | syrup / capsule | capsule / syrup |
| Formulation of product | "Echinaforce Hotdrink verum contains a hydroethanolic extract (65% v/v) of freshly harvested Echinacea purpurea. The tinctures from the herb (drug extraction ratio 1:12) and from the roots (drug extraction ratio 1:11) are combined at a ratio of 95% to 5%.Two hundred forty milligrams of the above active ingredient was concentrated to extractum spissum, which was supplemented with 276.5 mg Sambucus fructus succus recentis [fresh fruit juice] (elderberry), and excipients were added sufficient to give 1 mL Echinaforce Hotdrink. On analysis, the batch (No. 033492) was found to contain 883μg/100 mL dodecatetraenoic acid isobutylamide and101 mg/100 mL rutoside. "  Unsure if this is sambucus nigra (black elderberry) or other, given information from publication. However, currently available (2020) product information states this is black elderberry (https://www.avogel.co.uk/herbal-remedies/echinacea-echinaforce/hot-drink/). | 75 mg Tamiflu capsules |
| Standardization and manufacturing | "Echinafore Hotdrink [was] filled into 200-mL dark-brown glass bottles by A.Vogel Bioforce AG under good manufacturing practice conditions." The oseltamivir placebo "consisted of hard gelatine capsules filled with microcrystalline cellulose and were indistinguishable from verum capsules. "The [oseltamivir placebo was] manufactured under good manufacturing practice conditions and [was] batch released by Corden Pharma GmbH." | Oseltamivir verum was manufactured by "overencapsulation of original oseltamivir capsules (Tamiflu 75 mg, batch No. 01130082; Hoffmann-La Roche AG, Basel, Switzerland) using optically dense, dark green, hard gelatine capsules, size 0 (Capsugel, Bornem,Belgium). Corden Pharma GmbH (Plankstadt, Germany) manufactured comparator capsules packed in high-density polyethylene bottles each containing 10 capsules.""The Echinaforce Hotdrink placebo contained the same excipients as verum plus colorants and flavors (Günter Aroma GmbH, Beinwil, Switzerland) for masking (batch No. 033493)." |
| Dose of product | 5 mL syrup dissolved in approximately 150 mL hot water / 1 placebo capsule | 5 mL placebo syrup dissolved in approximately 150 mL hot water / 1 capsule |
| Frequency of dose | syrup 5 times/day for 3 days then 3 times/day for 7 days; capsule 2 times/day | capsule 2 times/day; syrup 5 times/day for 3 days then 3 times/day for 7 days |
| Duration of treatment | 10 days | 10 days |
| Concomitant medications | "Paracetamol and dextromethorphan were provided as rescue medication in form of Paralen 500 mg tablets and Stopex 30 mg tablets, respectively” | |

| **Rauš 2015** |  |  |
| --- | --- | --- |
| **Outcomes** |  |  |
| “Participants received a symptom diary to record their influenza symptoms in the morning and evening and throughout the study period or until recovery. Cough, nasal obstruction, sore throat, fatigue, headache, myalgia, feverishness, malaise, sweats, and/or chills were rated as 0=not present, 1=mild, 2=moderate, or 3=severe. Axillary body temperature was measured by electronic thermometer in degrees Celsius, sleeping disorders for the preceding night and ability to return to regular daily activities were scored yes/no, and intake of study medication as well as rescue and comedication and cotherapies was recorded. Occurrence of complications (eg, pneumonia, sinusitis, bronchitis, or other) was recorded at the close-out visit as was the need for intermed visits/contacts, hospitalization, and for antibiotic treatment. Patients and physicians rated tolerability as well as efficacy on a subjective basis using a Likert scale where 1=very good, 2=good, 3=moderate, and 4=poor. Finally, patients gave their opinions whether they would take the same medication again. The primary end point of the study was the cumulative proportion of patients with influenza symptoms alleviated (recovered) after 1 day, 5 days, and 10 days of treatment. Recovery was defined as the first day when cough, nasal obstruction, sore throat, fatigue, headache, myalgia, and feverishness were rated as absent or mild in the evening. The analysis of noninferiority between treatments based on the per-protocol cohort, defined as those who fulfilled inclusion and exclusion criteria, took at least 80% of study medication, and did not take unauthorized concomitant medication. Secondary variables included evaluation of further influenza symptoms, number of days with sleep disturbance, time point of return to normal activity, evolution of body temperature, use of rescue medication, and additional health care contacts. The proportion of patients experiencing respiratory respective gastrointestinal complications that required premature treatment stop was analyzed.” | | |

| **Tiralongo 2016** |
| --- |
| **Identification**  SPONSORSHIP SOURCE  "The study was funded by Iprona AG, Italy. The sponsor provided the elderberry and placebo capsules and was partially involved in the design of the study. The randomisation of participants, collection, analysis, interpretation and publication of the data was conducted by the researchers. The decision to publish the results was made by the researchers and supported by the sponsor."  COUNTRY  Australia  SETTING  Outpatient  COMMENTS  Year(s) conducted: 2013-2014  Prevention study for persons traveling overseas. Target is respiratory symptoms.  **Author's contact details**  NAME  Evelin Tiralongo  INSTITUTION  Griffith University  EMAIL  e.tiralongo@griffith.edu.au  ADDRESS  School of Pharmacy, Griffith University, Gold Coast campus, Queensland 4222, Australia  **COIs**  Authors did not disclose potential COIs |
| **Methods**  DESIGN  Randomized controlled trial, quadruple-blind  GROUP  Parallel group  HOW WERE ADVERSE EVENTS ASSESSED?  They do not report methods for assessing adverse events, however they do mention some that occurred in the treatment and placebo groups. |
| **Population**  INCLUSION CRITERIA  "Volunteers were included if they were a minimum of 18 years of age and in good general health."  EXCLUSION CRITERIA  "Volunteers were excluded if they were currently in another clinical trial or were in one less than 30 days ago, had a known plant allergy, were suffering from respiratory diseases (e.g., asthma, chronic obstructive pulmonary disease), had any other condition that could compromise the study or the participants health (e.g., autoimmune disease, cystic fibrosis), had received flu vaccination less than 10 days of starting the trial, were lactating, pregnant or planning to become pregnant or were on regular treatment with antibiotics, corticosteroids, antihistamines, antivirals, non-steroidal anti-inflammatory drugs, anticancer drugs and immune-suppressants."  GROUP DIFFERENCES  "Participants in placebo and active groups did not differ significantly at baseline." |

| **Tiralongo 2016** | | | |
| --- | --- | --- | --- |
| **Baseline Characteristics** |  |  |  |
| **Characteristic** | **Elderbery** | **Placebo** | **Overall** |
| Sample size | 158 | 154 | 312 |
| Age in years | Mean (sd) 52 (16) | Mean (sd) 50 (17) | Mean (sd) 51 (16) |
| Male/Female numbers | 50/108 | 56/98 | 106/206 |
| Race/Ethnicity (if reported) | NR | NR | NR |
| Socioeconomic status (if reported) | NR | NR | NR |
| Comorbidities and known risk factors:  number smokers  travel time >16h | 5  112 | 8  106 | 13  218 |
| Influenza A (n) | NA | NA | NA |
| Influenza B (n) | NA | NA | NA |
| Duration of illness at study entry (first treatment) (mean with units) | NA | NA | NA |
| Fever at study entry (n) | NA | NA | NA |
| Cough (any) at study entry (n) | NA | NA | NA |
| Dry cough at study entry (n) | NA | NA | NA |
| Productive cough at study entry (n) | NA | NA | NA |
| Headache at study entry (n) | NA | NA | NA |
| Nasal congestion at study entry (n) | NA | NA | NA |
| Muscle aches at study entry (n) | NA | NA | NA |
| Mucus discharge at study entry (n) | NA | NA | NA |
| Fatigue at study entry (n) | NA | NA | NA |
| Sore throat at study entry (n) | NA | NA | NA |

| **Tiralongo 2016** |  |  |
| --- | --- | --- |
| **Intervention(s) and Comparator(s)** |  |  |
| **Characteristic** | **Elderbery** | **Placebo** |
| Name of commercial product | Rubini (per reference 14) (produced by Iprona AG, Lana (BZ) Italy, under the BerryPharma brand | N/A |
| Type of product | Capsules | Capsules |
| Formulation of product | "300 mg of elderberry (Sambucus nigra L., Haschberg variety, Steiermark region in Austria) extract (22% polyphenols (i.e., quercetin and its glycosides, rutin), 15% anthocyains (i.e., cyanidin and pelargonidin glycosides) and 150 mg of rice flour. The 300 mg elderberry extract also contain several mineral, trace elements and vitamins including relatively high levels of magnesium 1.19 mg (Mg: 3.97 mg/g)."  Plant part from which elderberry was extracted is not specified. Berries are discussed in detail in Introduction so likely but not explicit that it was berries. | "Placebo capsules were manufactured to match the elderberry capsules in size, excipients and appearance." |
| Standardization and manufacturing | "The elderberry capsules used in this trial were produced by Plantafood in Germany in accordance with the principles and guidelines of Good Manufacturing Practice." | NR |
| Dose of product | 1 capsule | 1 capsule |
| Frequency of dose | 2 capsules/day beginning 10 days before travel and 3 capsules/day from 1 day before travel until 4-5 days after arriving at destination. | 2 capsules/day beginning 10 days before travel and 3 capsules/day from 1 day before travel until 4-5 days after arriving at destination |
| Duration of treatment | Total duration of treatment 15-16 days | Total duration of treatment 15-16 days |
| Concomitant medications | "In both groups half the participants with a defined cold used co-medication to relieve symptoms. On average participants co-medicated for 1.5 days; the difference was not significant between groups (p=0.9)." | |

| **Tiralongo 2016** |  |  |
| --- | --- | --- |
| **Outcomes** |  |  |
| “The main question the participant answered in the diary, “Do you believe you have a cold today?” was answered yes or no. During acute colds, the symptoms “headache”, “chilliness”, “sneezing”, “nasal obstruction”, “nasal discharge”, “sore throat”, “cough”, and “malaise” were rated on a 4-point Likert scale with 0 or no entry = absence, 1 = mild, 2 = moderate, and 3 = severe symptoms. In addition, the participant indicated in the diary the daily intake of concomitant medication and/or therapy.”  “The primary outcome measure for this study is the total number of cold episode days measured for the previous six days”. “[A] cold episode was defined as a minimal total symptom score of 14 (summed over a minimum of six consecutive days), and the participants believed they had a cold and/or reported rhinorrhoea that lasted for ≥ 3 days”. This matrix is based on definition by Jackson for the common cold as a clinical entity.  However, in the trial registration, the primary outcome was the daily assessment of the number of participants suffering from upper respiratory symptoms using the Jackson Score, and it is not clear whether this simply refers to participants with symptoms or to participants with defined cold episodes.  In addition, two quality of life instruments were used:  “[T]he upper respiratory symptom-related Quality of life (QoL) was measured using the questions from the 21-item Wisconsin Upper Respiratory Symptom Survey (WURSS-21)”  “The SF-21 was utilised to assess general Quality of Life (QoL).” (This appears to be the SF-12)  Finally, the Perceived Stress Scale (PSS) was used to identify a sub-group of participants with heightened stress and select them for analysis of a possible correlation between increased stress and the effects of elderberry. | | |

| **Zakay-Rones 1995** |
| --- |
| **Identification**  **Study details**  SPONSORSHIP SOURCE  Not mentioned  COUNTRY  Israel  SETTING  Outpatient  COMMENTS  Year(s) conducted: NR  Treatment study for persons with Influenza symptoms.  **Author's contact details**  NAME  Zichria Zakay-Rones  INSTITUTION  Department of Virology, Hebrew University-Hadassah Medical School  EMAIL  Not given  ADDRESS  Prof. Zichria Zakay-Rones Department of Virology Hebrew University-Hadassah Medical School FOB 12272 91120 Jerusalem, Israel  **COIs**  Not disclosed |
| **Methods**  DESIGN  Randomized controlled trial, double-blinded, as sub-study of an in vitro study on antiviral properties of elderberry extracts  GROUP  Parallel group  HOW WERE ADVERSE EVENTS ASSESSED?  "Before the beginning of the study, SAM was tested for the absence of side-effects on 35 healthy individuals from Jerusalem who received 4 tablespoons daily for 3 days. No side effects were recorded." No other mention of side effects or adverse events. |
| **Population**  INCLUSION CRITERIA  "Patients who were admitted to the study had at least three of the following symptoms of less than 24 h duration: fever >38°C, myalgia, nasal discharge, and cough." From the aims of the study, patients had "illness caused by influenza viruses" and were members of the "normally healthy population that was not previously vaccinated against flu."  EXCLUSION CRITERIA  "In the presence of streptococcus A (tested with Biosign strep. A, Princetown Biomeditech Corp., Princeton, NJ), patients with a sore throat were excluded from the study." After randomization, patients were omitted from analysis if they were negative in all virological tests.  GROUP DIFFERENCES  Group differences not explicitly assessed but appear reasonably similar (Table 3). |

| **Zakay-Rones 1995** | | | |
| --- | --- | --- | --- |
| **Baseline Characteristics** |  |  |  |
| **Characteristic** | **Elderbery** | **Placebo** | **Overall** |
| Sample size* | 15 | 12 | 27 |
| Age in years | Range 5-50 | Range 7-56 | Range 5-56 |
| Male/Female numbers | 9/6 | 9/3 | 18/9 |
| Race/Ethnicity (if reported) | NR | NR | NR |
| Socioeconomic status (if reported) | NR | NR | NR |
| Comorbidities and known risk factors: number smokers | NR | NR | NR |
| Influenza A (n) | 0 | 2 | 2 |
| Influenza B (n)** | 13 | 10 | 23 |
| Duration of illness at study entry (first treatment) (mean with units) | <24 hours | <24 hours | <24 hours |
| Fever at study entry (n) | NR | NR | 26*** |
| Cough (any) at study entry (n) | NR | NR | 5*** |
| Dry cough at study entry (n) | NR | NR | NR |
| Productive cough at study entry (n) | NR | NR | NR |
| Headache at study entry (n) | NR | NR | 27*** |
| Nasal congestion at study entry (n) | NR | NR | 23*** |
| Muscle aches at study entry (n) | NR | NR | 14*** |
| Mucus discharge at study entry (n) | NR | NR | NR |
| Fatigue at study entry (n) | NR | NR | 24*** |
| Sore throat at study entry (pharyngitis)(n) | NR | NR | 20*** |

*20 randomized to each treatment group however all data from baseline onwards is presented per protocol.

**In the elderberry treatment group, one participant was positive to respiratory syncytial virus (RSV) and one participant was positive to both Adenovirus and RSV.

***Calculated from percentages reported in study.

| **Zakay-Rones 1995** |  |  |
| --- | --- | --- |
| **Intervention(s) and Comparator(s)** |  |  |
| **Characteristic** | **Elderbery** | **Placebo** |
| Name of commercial product | Sambucol® | NA |
| Type of product | Syrup | Syrup |
| Formulation of product | “A standardized extract, Sambucol® (SAM), is a preparation based on the berries of the black elder, used as herbal remedy against influenza virus infections. It contains a high amount of three flavonoids (Bronnum-Hansen and Hansen, 1983).” "Sambucol® (Razei Bar Ltd, Jerusalem) is a syrup containing elderberry juice, raspberry extract, glucose, citric acid, and honey.” | Composition of placebo not described. |
| Standardization and manufacturing | Not described | Not described |
| Dose of product | 1 tablespoon | 1 tablespoon |
| Frequency of dose | 2 times/day for children; 4 times/day for adults | 2 times/day for children; 4 times/day for adults |
| Duration of treatment | 3 days | 3 days |
| Concomitant medications | Not described | |

| **Zakay-Rones 1995** |  |  |
| --- | --- | --- |
| **Outcomes** |  |  |
| Persistence of and improvement in symptoms and assessment of complete cure was checked daily. Mean number of days with specific symptoms, percentage of participants with symptoms, cure rate and overall duration of illness was evaluated. | | |

| **Zakay-Rones 2004** |
| --- |
| **Identification**  Study details  SPONSORSHIP SOURCE  "The study was sponsored by Razei Bar, Jerusalem, Israel."  COUNTRY  Israel  SETTING  Outpatient  COMMENTS  Year(s) conducted: 1999-2000  Treatment study for persons with Influenza A or B infections.  Author's contact details  NAME  First author: Z. Zakay-Rones; Corresponding author: Erling Thom  INSTITUTION  First author: Department of Virology, Hebrew University-Hadassah Medical School; Corresponding author: PAREXEL Norway AS  EMAIL  erling.thom@parexel.com  ADDRESS  PO Box 210, N-2001 Lillestrøm, Norway  **COIs**  Not disclosed |
| **Methods**  DESIGN  Randomized controlled trial, placebo-controlled  GROUP  Parallel group  HOW WERE ADVERSE EVENTS ASSESSED?  "As sedation is a main side-effect of most anti-influenza medications, the participants were specifically asked if they had any problem with sedation during the study period." |
| **Population**  INCLUSION CRITERIA  "80 candidates presenting at an investigator’s office with respiratory influenza symptoms (classified as 487 in the International Classification of Primary Care) were screened for inclusion. Those with verified influenza were enrolled in the study. All subjects had a fever ≥38.0 °C and at least one respiratory influenza symptom." "The subjects were all healthy individuals, with the exception of the current episode of influenza, and did not belong to high-risk groups." (unclear whether the latter are inclusion criteria or purely descriptive)  EXCLUSION CRITERIA  "Exclusion criteria included those who were pregnant or breastfeeding, those with suspected bacterial infections, recent antiviral therapy, recent participation in another clinical trial, anti-influenza vaccination and treatment for chronic diseases"  GROUP DIFFERENCES  No important group differences (Tables 1 and 2) as follows: "At the beginning of the study, no significant differences were observed between the active treatment group (those receiving elderberry syrup; n= 30) and the placebo group (n= 30) with regard to demographic characteristics, smoking status, clinical symptoms, problems related to sleeping and normal activity or absenteeism from work. The mean duration of the illness before receiving the first dose was 27.2 h.The baseline (day 1) VAS scores for the different parameters examined are listed inTable 2. There were no significant differences between the groups." |

| **Zakay-Rones 2004** | | | |
| --- | --- | --- | --- |
| **Baseline Characteristics** |  |  |  |
| **Characteristic** | **Elderbery** | **Placebo** | **Overall** |
| Sample size | 30 | 30 | 60 |
| Age in years | Mean (sd) 30.6 ± 2.9 | Mean (sd) 29.4 ± 2.8 | Mean (sd) 30.0 ± 2.9* (range 18 -54) |
| Male/Female (n) | 18/12 | 15/15 | 33/27* |
| Race/Ethnicity (if reported) (n) | NR | NR | NR |
| Socioeconomic status (if reported) (n) | NR | NR | NR |
| Comorbidities and known risk factors: number smokers | 4 | 1 | 5 |
| Influenza A (n) | 26 | 28 | 54 |
| Influenza B (n) | 4 | 2 | 6 |
| Duration of illness at study entry (first treatment) (mean with units) | NR | NR | 27.2 hours |
| Fever at study entry (n) | 30 | 30 | 60 |
| Cough (any) at study entry (n) | 30 | 30 | NR |
| Dry cough at study entry (n) | 5 | 7 | 12 |
| Productive cough at study entry (n) | 25 | 23 | 48 |
| Headache at study entry (n) | NR | NR | NR |
| Nasal congestion at study entry (n) | NR | NR | NR |
| Muscle aches at study entry (n) | NR | NR | NR |
| Mucus discharge at study entry (n) | NR | NR | NR |
| Fatigue at study entry (n) | NR | NR | NR |
| Sore throat at study entry (n) | NR | NR | NR |

*calculated from reported data

| **Zakay-Rones 2004** |  |  |
| --- | --- | --- |
| **Intervention(s) and Comparator(s)** |  |  |
| **Characteristic** | **Elderbery** | **Placebo** |
| Name of commercial product | Sambucol® | NA |
| Type of product | Syrup | Syrup |
| Formulation of product | "A standardized elderberry extract (Sambucol®, Razei Bar, Jerusalem, Israel) was used. The syrup formulation contained 38% of the standardized extract plus small amounts of raspberry extract, glucose, citric acid and honey."  We know from Zakay-Rones 1995 that this is black elderberry, but it’s not specified in this article. Also, introduction/discussion allude to berries as the part of the elderberry plant responsible for constituents/the active intervention, but the part of the plant used in intervention is not directly specified in this article. We know from Zakay-Rones 1995 that Sambucol is extract from berries. | "The placebo syrup did not contain the elderberry extract, but was otherwise identical." |
| Standardization and manufacturing | "Standardization of the flavonoid content was maintained by ensuring the absorbance at 516 nm was above 0.60. The extract is produced according to good manufacturing practice, and both its production and the production facilities are certified by the Israeli Health Authorities." "Both syrups were produced and supplied by Razei Bar Ltd (Jerusalem, Israel)" | "Both syrups were produced and supplied by Razei Bar Ltd (Jerusalem, Israel)" |
| Dose of product | 15 ml syrup | 15 ml syrup |
| Frequency of dose | 4 times per day with meals | 4 times per day with meals |
| Duration of treatment | 5 days | 5 days |
| Concomitant medications | "Patients were allowed to take concomitant medications during the study in the form of the antipyretic/analgesic agent paracetamol (Paracet®, Weifa, Oslo, Norway; 500 mg tablets) and/or a dose-metered nasal spray (Otrivin®, Novartis, Basel, Switzerland; 1 mg/ml) to relieve the influenza symptoms(rescue medications) if treatment with Sambucol®or placebo did not help. These medications were provided free of charge and were marketed drugs. In cases of known allergy to the rescue medications, alternatives were provided (acetylsalicylic pain killers instead of paracetamol and salt water spray instead of Otrivin®). Patients recorded the date, time and dose of any concomitant medication taken, as well as the name of the drug used." | |

| **Zakay-Rones 2004** |  |  |
| --- | --- | --- |
| **Outcomes** |  |  |
| Self-assessed VAS scale (from 0 to 10 where 10 is worse symptom) for global evaluation of well-being and for aches and pains, degree of coughing, frequency of coughing, quality of sleep, mucus discharge in the respiratory tract and nasal congestion. “Patients scored their symptoms on diary cards at baseline, four times a day during treatment and twice daily for 5 days after the treatment had finished.” | | |
