# Supplementary material for: Elderberry for prevention and treatment of viral respiratory illnesses: a systematic review
Source: BMC Complement Med Ther. 2021 Apr 7;21:112. doi: 10.1186/s12906-021-03283-5 (PMC8026097; doi:10.1186/s12906-021-03283-5)
Supplement: Supplementary file 4 — Additional file 4. [file 12906_2021_3283_MOESM4_ESM.docx]

| Comparison/Outcome | Results | Downgrade for risk of bias? | | Downgrade for inconsistency? (e.g, I^2^≥50%) | | Downgrade for indirectness? (Review and study PICO do not match) | | Downgrade for imprecision? (total n<400, or CI includes no important effect and SMD of ±0.5) | | Downgrade for other reasons? (e.g., publication bias) | | GRADE certainty of evidence |
| --- | --- | --- | --- | --- | --- | --- | --- | --- | --- | --- | --- | --- |
| **Elderberry for prevention of viral respiratory illness** | | | | | | | | | | | | |
| Elderberry vs Placebo/# participants developing a cold | RR 0.69 [0.34, 1.39]  I^2^=0% 1 study 312 participants [29 events] | -1 | 0 | | 0 | | -1 | | 0 | | Low | |
| Elderberry vs Placebo/days to resolution of cold | MD -2.13 [-4.16, -0.10]  I^2^=0% 1 study 29 participants | -1 | 0 | | 0 | | -2 | | 0 | | Very low | |
| Elderberry vs Placebo/severity of cold | MD -13.69 [-24.54, -2.84] I^2^=0% 1 study 29 participants | -1 | 0 | | 0 | | -2 | | 0 | | Very low | |
| Elderberry vs Placebo/Adverse events | RR 0.65 [0.11, 3.84]  I^2^=0% 1 study 312 participants [5 events] | -1 | 0 | | 0 | | -2 | | 0 | | Very low | |
| **Elderberry for treatment of viral respiratory illness** | | | | | | | | | | | | |
| Elderberry vs Placebo/# participants with complete cure at 2 days | RR 2.40 [0.59, 9.82]  I^2^=0% 1 study 27 participants | -1 | 0 | | 0 | | -2 | | 0 | | Very low | |
| Elderberry vs Placebo/# participants with complete cure at 3 days | RR 2.60 [1.14, 5.93]  I^2^=0% 1 study 27 participants | -1 | 0 | | 0 | | -2 | | 0 | | Very low | |
| Elderberry vs Placebo/# participants with complete cure at 4 days | RR 1.94 [1.12, 3.36]  I^2^=0% 1 study 27 participants | -1 | 0 | | 0 | | -2 | | 0 | | Very low | |
| Elderberry vs Placebo/time to global improvement or cure/ | MD -2.68 [-5.23, -0.13]  I^2^=94% 2 studies 87 participants | -1 | -2 | | 0 | | -1 | | 0 | | Very low | |
| Elderberry vs Placebo/Any adverse event | RR not estimable 2 studies 124 participants | - | - | | - | | - | | - | | Unable to estimate | |
| **Elderberry-containing mixed product for treatment of viral respiratory illness** | | | | | | | | | | | | |
| Echinaforce + Placebo Oseltamivir vs Oseltamivir + Placebo Echinaforce/  # participants recovered at 1 day | RR 0.36 [0.10, 1.30]  I^2^=0% 1 study 420 participants [12 events] | -1 | 0 | | 0 | | -1 | | 0 | | Low | |
| Echinaforce + Placebo Oseltamivir vs Oseltamivir + Placebo Echinaforce/  # participants recovered at 5 days | RR 1.03 [0.85, 1.25]  I^2^=0% 1 study 420 participants [208 events] | -1 | 0 | | 0 | | -1 | | 0 | | Low | |
| Echinaforce + Placebo Oseltamivir vs Oseltamivir + Placebo Echinaforce/  # participants recovered at 10 days | RR 1.06 [0.99, 1.14]  I^2^=0% 1 study 420 participants [369 events] | -1 | 0 | | 0 | | 0 | | 0 | | Moderate | |
| Echinaforce + Placebo Oseltamivir vs Oseltamivir + Placebo Echinaforce/  # participants with complications 10 days | RR 0.38 [0.14, 1.04]  I^2^=0% 1 study 420 participants [19 events] | -1 | 0 | | 0 | | -1 | | 0 | | Low | |
| Echinaforce + Placebo Oseltamivir vs Oseltamivir + Placebo Echinaforce/  Any adverse event | RR 0.82 [0.51, 1.33  I^2^=0% 1 study 460 participants [58 events] | -1 | 0 | | 0 | | -1 | | 0 | | Low | |
