# Supplementary material for: Elderberry for prevention and treatment of viral respiratory illnesses: a systematic review
Source: BMC Complement Med Ther. 2021 Apr 7;21:112. doi: 10.1186/s12906-021-03283-5 (PMC8026097; doi:10.1186/s12906-021-03283-5)

1. Improvement in overall influenza symptoms


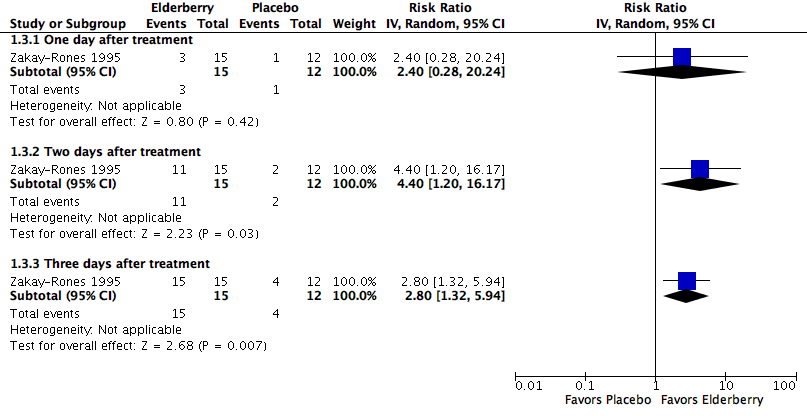


2. Complete cure of influenza symptoms


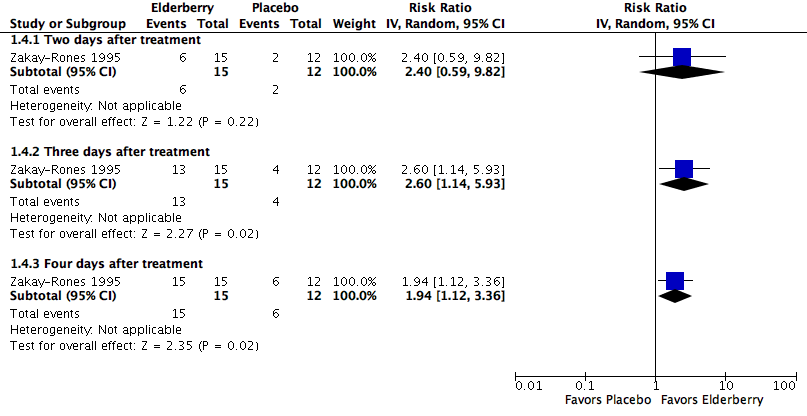


3. Resolution of influenza symptoms at 48 hours


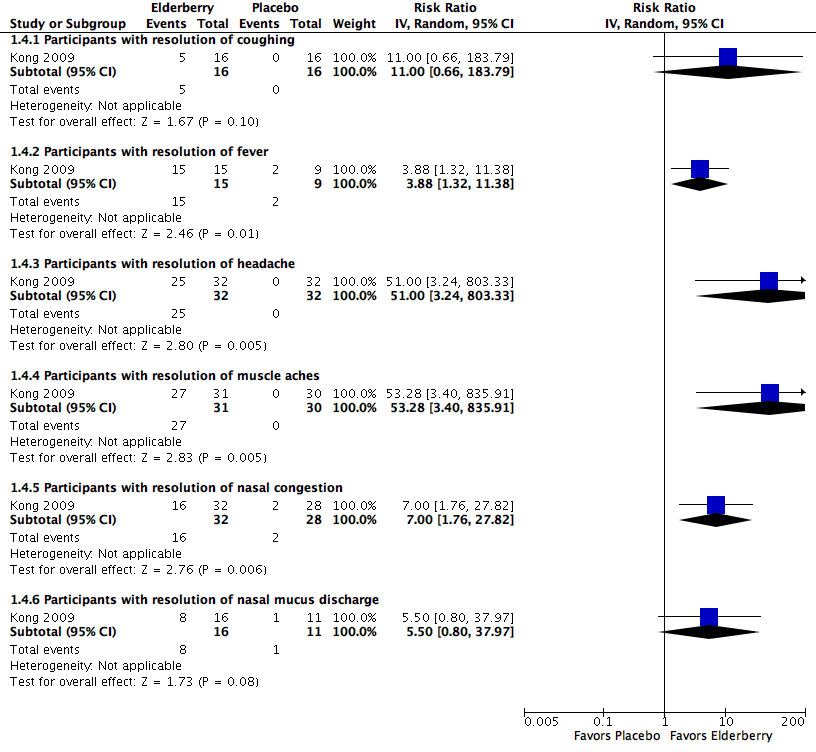


4. Duration of influenza symptoms (fever)


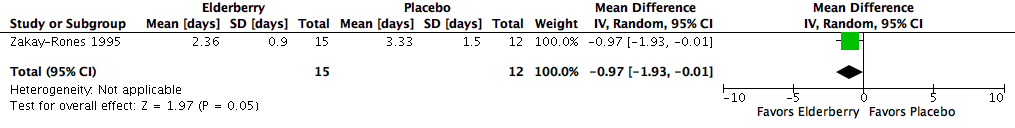


5. Severity of influenza symptoms at 48 hours


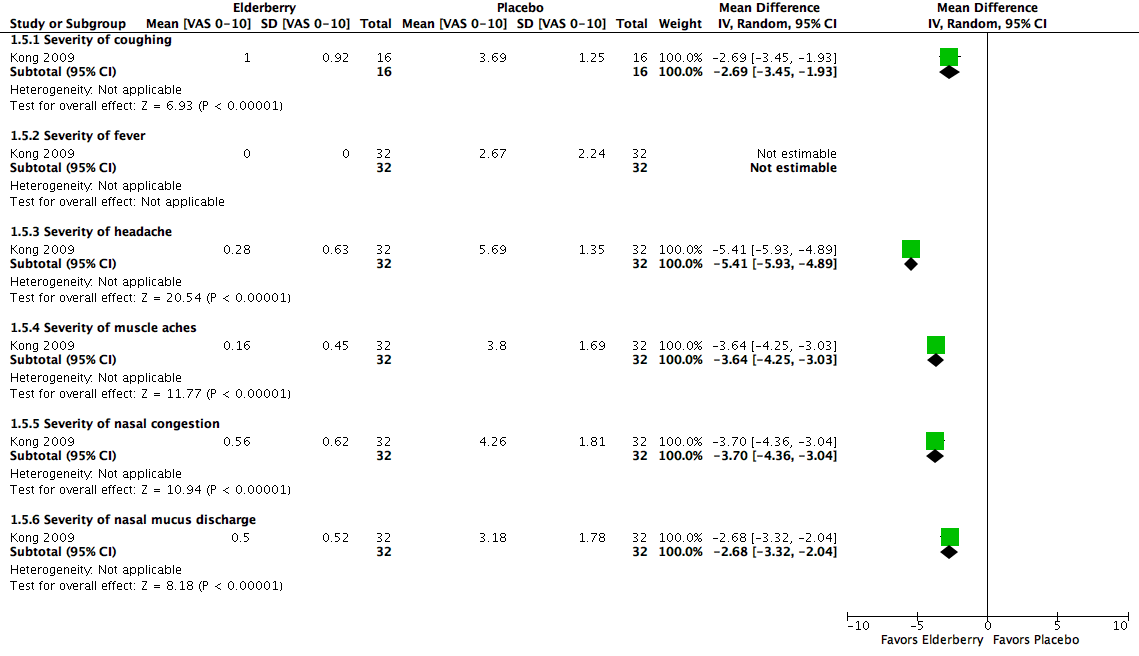

Supplement: Supplementary file 5 — Additional file 5. [file 12906_2021_3283_MOESM5_ESM.docx]
